# Cortical Overexpression of Neuronal Calcium Sensor-1 Induces Functional Plasticity in Spinal Cord Following Unilateral Pyramidal Tract Injury in Rat

**DOI:** 10.1371/journal.pbio.1000399

**Published:** 2010-06-22

**Authors:** Ping K. Yip, Liang-Fong Wong, Thomas A. Sears, Rafael J. Yáñez-Muñoz, Stephen B. McMahon

**Affiliations:** 1 Neurorestoration Group, Wolfson CARD, King's College London, Guy's Campus, London, United Kingdom; 2 Henry Wellcome LINE, Dorothy Hodgkin Building, Bristol University, Bristol, United Kingdom; 3 School of Biological Sciences, Royal Holloway-University of London, Egham, Surrey, United Kingdom; University of California San Francisco, United States of America

## Abstract

Following trauma of the adult brain or spinal cord the injured axons of central neurons fail to regenerate or if intact display only limited anatomical plasticity through sprouting. Adult cortical neurons forming the corticospinal tract (CST) normally have low levels of the neuronal calcium sensor-1 (NCS1) protein. In primary cultured adult cortical neurons, the lentivector-induced overexpression of NCS1 induces neurite sprouting associated with increased phospho-Akt levels. When the PI3K/Akt signalling pathway was pharmacologically inhibited the NCS1-induced neurite sprouting was abolished. The overexpression of NCS1 in uninjured corticospinal neurons exhibited axonal sprouting across the midline into the CST-denervated side of the spinal cord following unilateral pyramidotomy. Improved forelimb function was demonstrated behaviourally and electrophysiologically. In injured corticospinal neurons, overexpression of NCS1 induced axonal sprouting and regeneration and also neuroprotection. These findings demonstrate that increasing the levels of intracellular NCS1 in injured and uninjured central neurons enhances their intrinsic anatomical plasticity within the injured adult central nervous system.

## Abstract 2

Overexpression of neuronal calcium sensor 1 in cortical neurons can help restore axonal plasticity and regeneration following axonal injury in adult rats, and can also improve behavioral function.

## Author Summary

Following trauma to the central nervous system (brain or spinal cord), neurons show very little capacity to re-grow their axons, which can lead to a permanent loss of function in those regions. In this study, we show that this failure of axon re-growth is associated with low intracellular levels of a small molecule called neuronal calcium sensor-1 (NCS1). We modified a non-replicating virus in two ways so as to increase the level of NCS1 in neurons while simultaneously labelling them with a green fluorescent protein, which allowed us to track neuronal growth. Using this virus to increase the level of NCS1 in a particular group of neurons that communicate between the brain and spinal cord, we showed that new axonal growth occurred in the spinal cord with or without injury to the neurons. Electrophysiological assessments demonstrated that these new processes formed functional connections in the spinal cord, and behavioural experiments revealed that this recovery also helped the animals move their limbs more effectively. Furthermore, an increase in NCS1 protected these neurons, such that fewer of them died after the injury. These findings demonstrate that increasing the intracellular levels of NCS1 in neurons can aid in the recovery from central nervous system injury, and can help improve behavioural function.

## Introduction

Spinal cord injury is a significant clinical problem that produces life long disability, although in a minority of patients some degree of recovery can occur spontaneously without any therapeutic intervention [1],[2]. There are several possible mechanisms that could be responsible for this, one being anatomical plasticity, but such plasticity is very limited [3]–[5].

There is a growing literature suggesting pharmacological interventions can enhance both axonal regeneration [6]–[9] and anatomical plasticity [10]–[14] within the spinal cord, but little is known about the intracellular mechanisms responsible for such plasticity.

Recently, we have found that following injury, the lentiviral overexpression of retinoic acid receptor β2 (RARβ2) induces regeneration in sensory and central axons [15],[16]. Microarray analysis of CNS tissue transduced with overexpressing RARβ2 lentivector was carried out to identify the intracellular molecular pathways involved in such regeneration. In unpublished data, this analysis revealed a significant upregulation of neuronal calcium sensor-1 (NCS1) in the transduced tissue as confirmed immunohistochemically and by real-time PCR.

NCS1 is highly conserved across species and emerges as a key intracellular calcium signalling component in a number of regulatory pathways in neurons [17],[18]. This small molecule has been implicated in neuronal survival [19], short-term synaptic plasticity [20], and enhanced synapse formation and transmission [21]. Recently, it has also been suggested to regulate neurite outgrowth in pond snails [22],[23] and in primary cultured embryonic chick dorsal root ganglia neurons [24].

Here we show using lentiviral vectors that NCS1 overexpressed in primary cultured adult cortical neurons increases neurite sprouting. Following corticospinal tract (CST) denervation by unilateral pyramidotomy, axons of uninjured corticospinal neurons (CSN) overexpressing NCS1 sprout across the midline to form functional connections in the CST-denervated spinal cord. In axotomized CSN, overexpression of NCS1 induces axonal sprouting and regeneration and also neuroprotection. These studies reveal NCS1 as an important intracellular molecule for promoting anatomical plasticity following CNS injuries in the adult.

## Results

### NCS1 Overexpression Induces Neurite Sprouting in Adult Rat Cortical Neurons In Vitro

**Figure 1:**
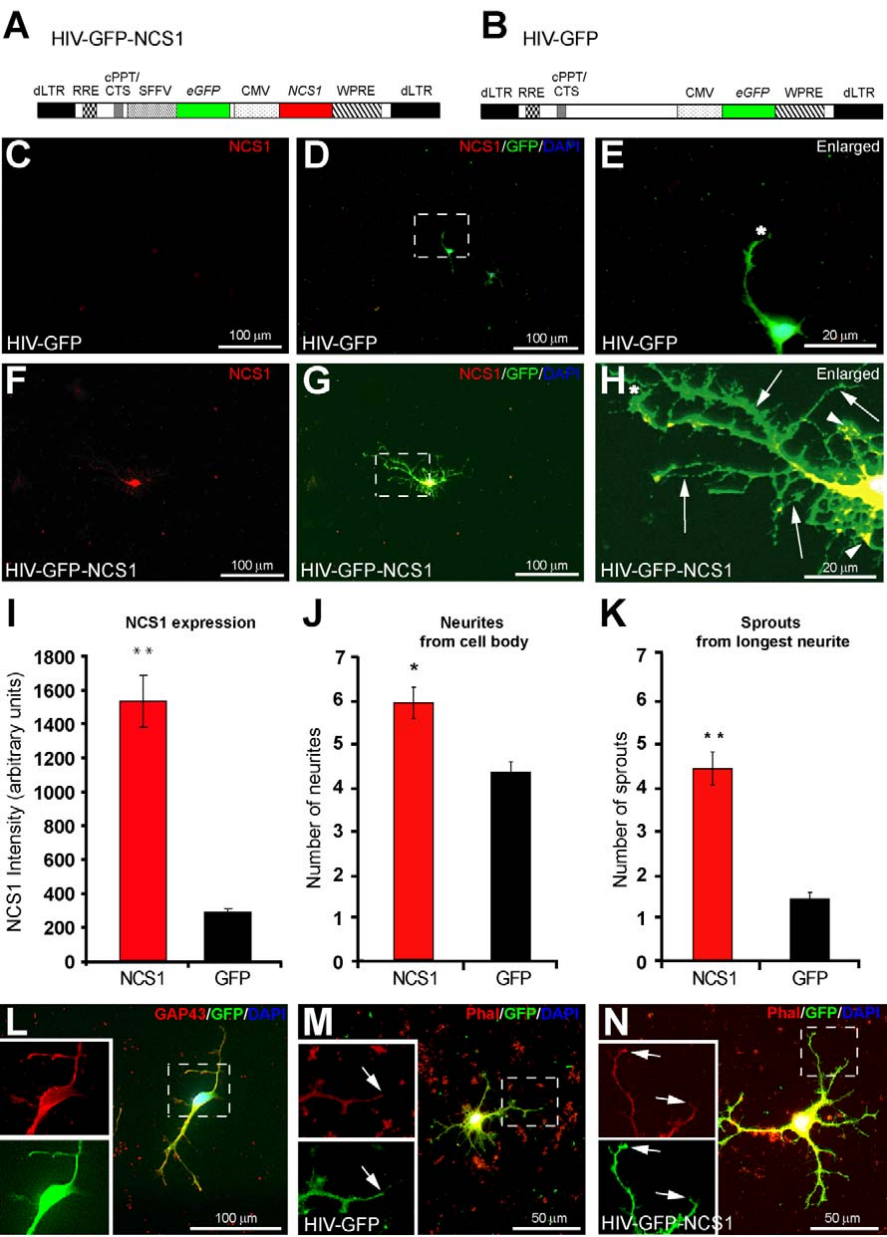
NCS1 overexpression promotes neurite sprouting in vitro. (A) Schematic vector map illustrating the HIV-GFP-NCS1 vector showing *eGFP* and *NCS1* gene driven by the SFFV and CMV promoter, respectively. (B) The control HIV-GFP vector contains only the *eGFP* gene driven by the CMV promoter. The lentiviral vector also contains: a self-inactivating 5′-deleted LTR (dLTR), a woodchuck hepatitis virus post-transcriptional regulatory element (WPRE), a central polypurine tract-central termination sequence (cPPT/CTS), and Rev response elements (RRE). (C–E) Primary culture of adult cortical neurons transduced with control vector HIV-GFP at a multiplicity of infection (MOI) of 10 showing low levels of NCS1 and limited neurite sprouting after 3 d in vitro (NCS1, red; GFP, green; DAPI, blue; longest neurite, *). (F–H) Transduction with HIV-GFP-NCS1 at a MOI of 10 induces extensive neurite sprouts from cell body and longest neurite (NCS1, red; GFP, green; DAPI, blue; longest neurite, *; neurites from cell body, arrowheads; sprouts from longest neurite, arrows). Panels E and H are higher power images of dashed boxes in panels D and G, respectively. (I) Significant elevated NCS1 levels were present in NCS1-transduced neurons compared to the controls. (J–K) A significant increase in neurite sprouting from both the cell bodies and longest neurites in NCS1- (red bar) compared with GFP- (black bar) transduced neurons was observed. (L) Dual immunolabelling of GFP positive (green) and the neuronal growth associated marker (GAP43) (red) in transduced neurons indicating cortical neurons. (M–N) Dual labelling of phalloidin staining (red) coexpressing with GFP immunostaining (green) indicates GFP driven by either SFFV or CMV promoter is a sufficient marker to distinguish neurites (arrows) from both NCS1- and control-transduced neurons, respectively. Data are expressed as mean ± SEM from *n* = 3 independent experiments. * *p*<0.05, ** *p*<0.01, Student's *t* test. Scale bars: (C,D,F,G,L) 100 µm, (M,N) 50 µm, and (E,H) 20 µm.

To transduce adult cortical neurons with NCS1 at high efficiency and to enable visualisation with an extrinsic marker, we constructed a minimal human immunodeficiency virus (HIV) based lentiviral vector expressing NCS1 and GFP under cytomegalovirus (CMV) and spleen focus-forming virus (SFFV) promoters, respectively, termed HIV-GFP-NCS1 (Figure 1A). A control vector termed HIV-GFP was used that expressed only GFP under the CMV promoter (Figure 1B). It has been shown that although CMV is a stronger promoter than SFFV in GFP expression, the percentage of transduced neurons with GFP expression was similar [25]. The level of NCS1 in transduced primary adult rat cortical neurons measured immunocytochemically was more than 5-fold greater than in the control HIV-GFP-transduced neurons (Figure 1C–1I). In control-transduced neurons, few sprouts were observed from cell bodies or neurites (Figures 1C–1E, 1J–1K, and S1). In contrast, neurons transduced with HIV-GFP-NCS1 showed significant increases in the numbers of sprouts from both cell bodies and neurites (Figures 1F–1H, 1J–1K, and S1). The GFP immunostained cortical cells were confirmed as neurons by co-immunopositive staining with the neuronal growth associated marker GAP43 (Figure 1L). Furthermore, although two different promoters to drive GFP were used, the adequacy of GFP immunostaining for neurite distribution in both the NCS1- and GFP-transduced groups was confirmed to be similar to that obtained with phalloidin staining (Figure 1M–1N).

**Figure 2:**
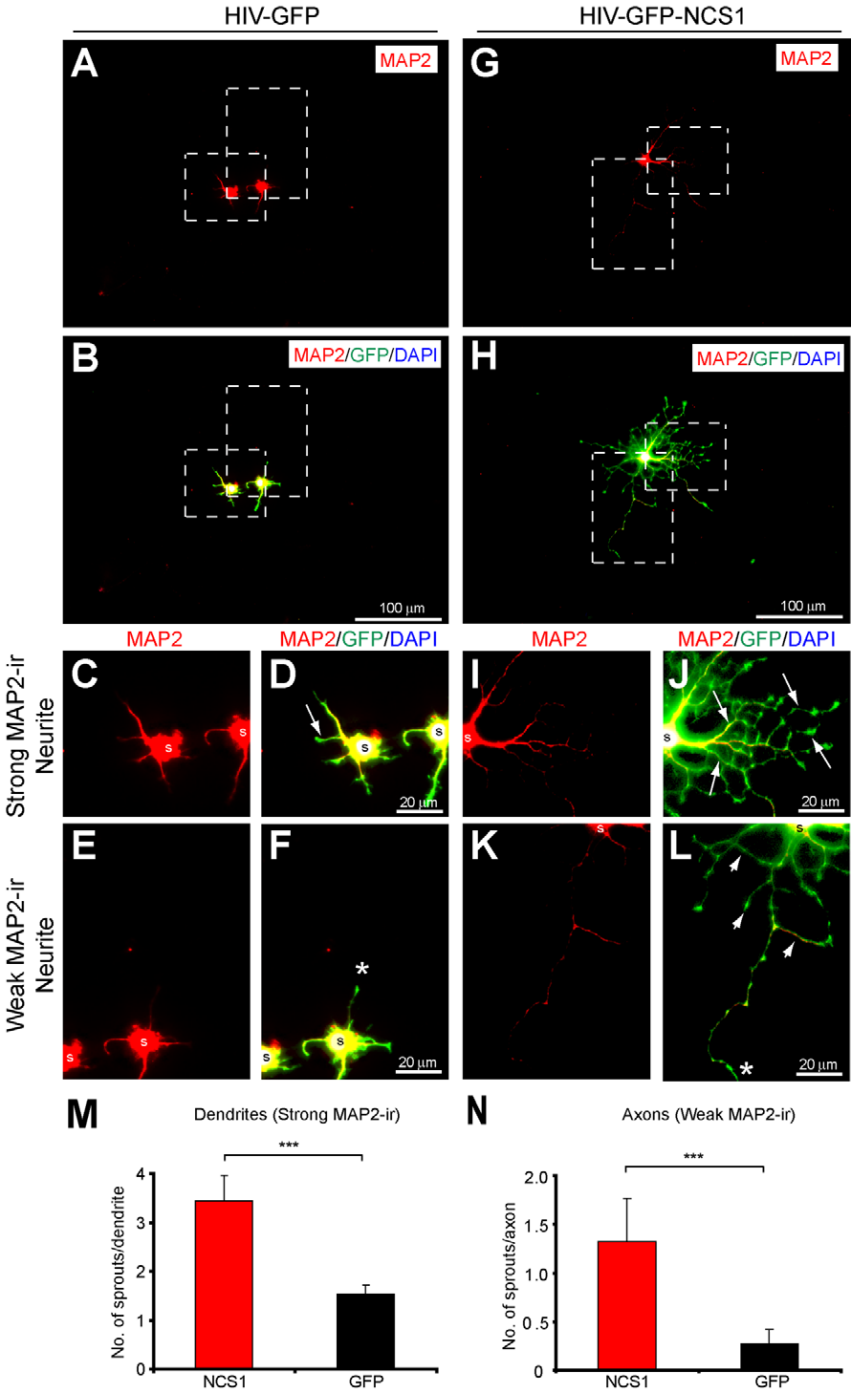
NCS1 overexpression induces sprouting in dendrites and axons in vitro. Primary cultured adult cortical neurons transduced with lentiviral vector were immunostained with the selective dendritic marker MAP2. Neurites with strong and weak MAP2 immunoreactivity were determined as dendrites and axons, respectively (MAP2, red; GFP, green; DAPI, blue; axon, *; soma, s). (A–B) Cortical neurons transduced with control HIV-GFP exhibited limited sprouting from short dendrites (C–D) and axons (E–F) after 3 d in vitro. (G–H) Transduction of cortical neurons with HIV-GFP-NCS1 induces extensive sprouting from long dendrites (I–J, arrows) and axons (K–L, arrowheads). (M–N) A significant increase in the number of sprouts from dendrites and axons was observed in NCS1- (red bar) compared with GFP- (black bar) transduced neurons. Panels C–F and I–L are higher power images of dashed boxes in panels A–B and G–H, respectively. Data are expressed as mean ± SEM from *n* = 3–4 independent experiments. *** *p*<0.001, Student's *t* test. Scale bars: (A–B and G–H) 100 µm and (C–F and I–L) 20 µm.

The type of neurite that has undergone sprouting with NCS1 overexpression was further investigated using the specific dendritic immunomarker microtubule-associated protein 2 (MAP2). MAP2 has been previously shown strongly and weakly to immunolabel dendrites and axons, respectively [26],[27]. In control HIV-GFP-transduced neurons, few sprouts were observed in both dendrites and axons (Figure 2A–2F). In contrast, a significant increase in the number of sprouts on both dendrites and axons was observed in HIV-GFP-NCS1-transduced neurons (Figure 2G–2N).

These data indicate that primary cultured adult cortical neurons overexpressed with NCS1 have significantly more neurites and sprouts from dendrites and axons than the control neurons.

### NCS1 Overexpression Induces Neurite Sprouting Via Akt Activation

**Figure 3:**
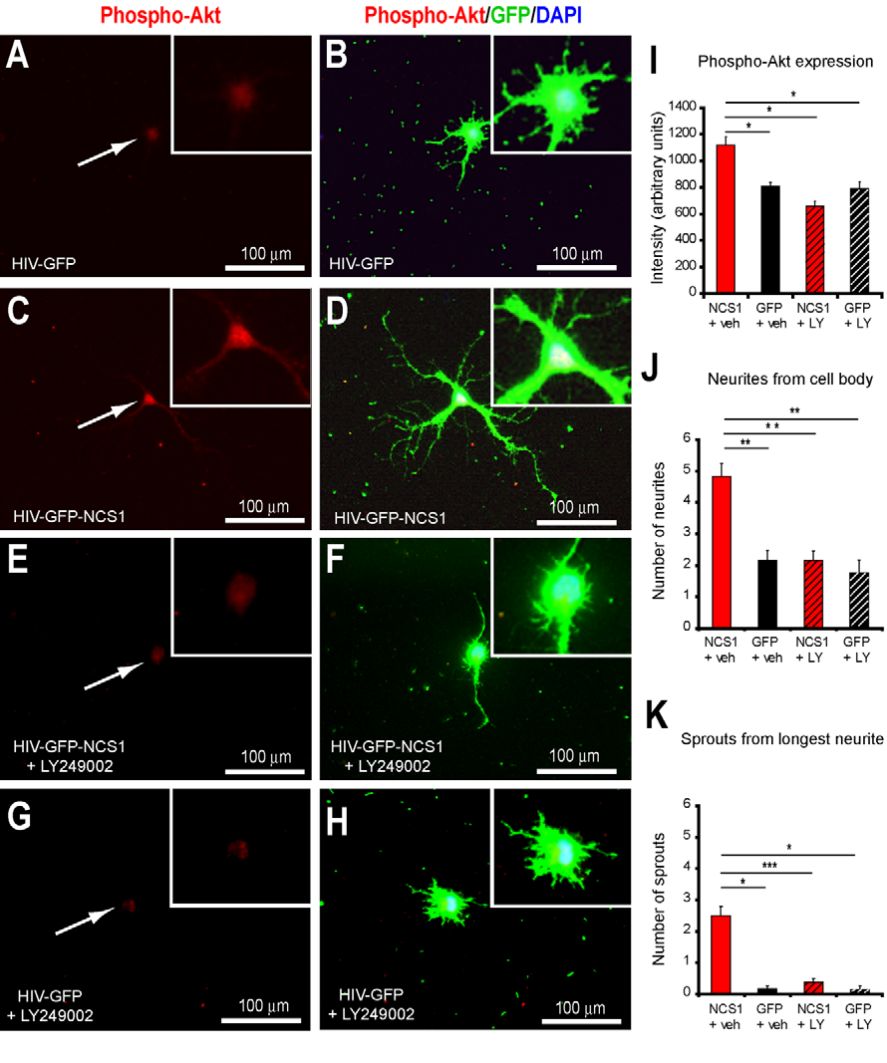
NCS1 overexpression induces neurite sprouting via Akt activation in vitro. (A–B) Primary cultured adult cortical neurons transduced with control HIV-GFP have low phospho-Akt levels (Phospho-Akt, red; GFP, green; DAPI, blue). (C–D) Transduction of cortical neurons with HIV-GFP-NCS1 induces high phospho-Akt levels (Phospho-Akt, red; GFP, green; DAPI, blue). (E–H) In the presence of the PI3K/Akt inhibitor LY249002, low phospho-Akt levels and reduced neurite sprouting were present in NCS1-transduced neurons, but LY249002 affected neither in control neurons. (I) Significant elevated phospho-Akt levels were present only in NCS1-transduced neurons in the absence of LY249002. (J–K) A significant fewer neurite sprouts from cell body and longest neurite was observed in the presence of LY249002 compared to vehicle treated NCS1-transduced neurons. NCS1-transduced neurons with vehicle (red bar), GFP-transduced neurons with vehicle (black bar), NCS1-transduced neurons with LY249002 (striped red bar), and GFP-transduced neurons with LY249002 (striped black bar). Data are expressed as mean ± SEM from *n* = 3–4 independent experiments. * *p*<0.05, ** *p*<0.01, *** *p*<0.001, Student's *t* test. Inserts are higher magnification of corresponding panels. Scale bars: 100 µm.

NCS1 has been shown to induce neuronal survival via the activation of the PI3K/Akt pathway [19]. We investigated whether this downstream intracellular pathway was also involved in the NCS1-induced neurite sprouting in primary cultures of adult mammalian neurons. The level of phospho-Akt in NCS1-transduced cortical neurons was significantly higher than in the control GFP-transduced group (Figures 3A–3D, 3I, and S2). Blockade of PI3K/Akt pathway with the inhibitor LY294002 caused a significant decrease in levels of phospho-Akt in the NCS1-transduced cortical neurons (Figures 3E–3F, 3I, and S3). This decrease corresponded to a significant 2-fold reduction in number of neurites from cell bodies and a 5-fold reduction in sprouts from neurites (Figure 3A–3K). However, neither phospho-Akt expression nor neurite sprout number was significantly changed in GFP-transduced neurons treated with LY249002 compared to vehicle treatment (Figures 3G–3K and S3).

These data indicate the levels of phospho-Akt are elevated in neurons with overexpressed NCS1 and blockade of Akt production reduces neurite sprouting in these neurons.

**Figure 4:**
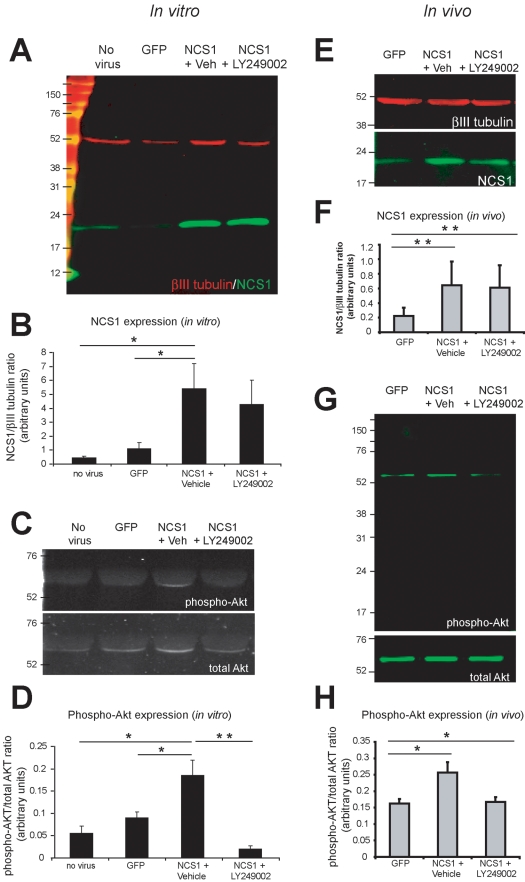
Western blots of elevated NCS1 and phospho-Akt in HIV-GFP-NCS1-transduced adult cortical neurons in vitro and in vivo. (A–B) The levels of NCS1 were significantly upregulated after transduction with HIV-GFP-NCS1 compared to the untransduced or control HIV-GFP-transduced neurons in vitro, with this increase unaffected by LY249002. (C–D) The significant upregulation of NCS1 level was associated with a corresponding significant increase in phospho-Akt levels in NCS1-transduced neurons compared to the controls in vitro. However, this increase in phospho-Akt levels was all but abolished in the presence of LY249002 in NCS1-transduced neurons. (E–H) After intracortical injections of HIV-GFP-NCS1, a significant increase in NCS1 and phospho-Akt was observed in the cortex of these rats compared to the control rats or NCS1-transduced rats in the presence of LY249002. Data are expressed as mean ± SEM from 3–4 animals. * *p*<0.05, ** *p*<0.01, Student's *t* test.

In Western blots, the levels of NCS1 were significantly higher in cortical neurons transduced with HIV-GFP-NCS1 compared with the controls both in vitro and in vivo (Figure 4A–4B, 4E–4F). This increase in NCS1 corresponds with a significant increase in phospho-Akt levels (Figure 4C–4D, 4G–4H). In the presence of LY249002, no significant change in NCS1 level occurred in NCS1-transduced cortical neurons compared to the controls (Figure 4A–4B, 4E–4F). This demonstrates that the reduction in phospho-Akt level was a direct result of LY249002 and not via the NCS1 overexpression itself. These data show that the neurite sprouting induced by NCS1 was indeed via the PI3K/Akt pathway.

### NCS1 Overexpression in CSN In Vivo

**Figure 5:**
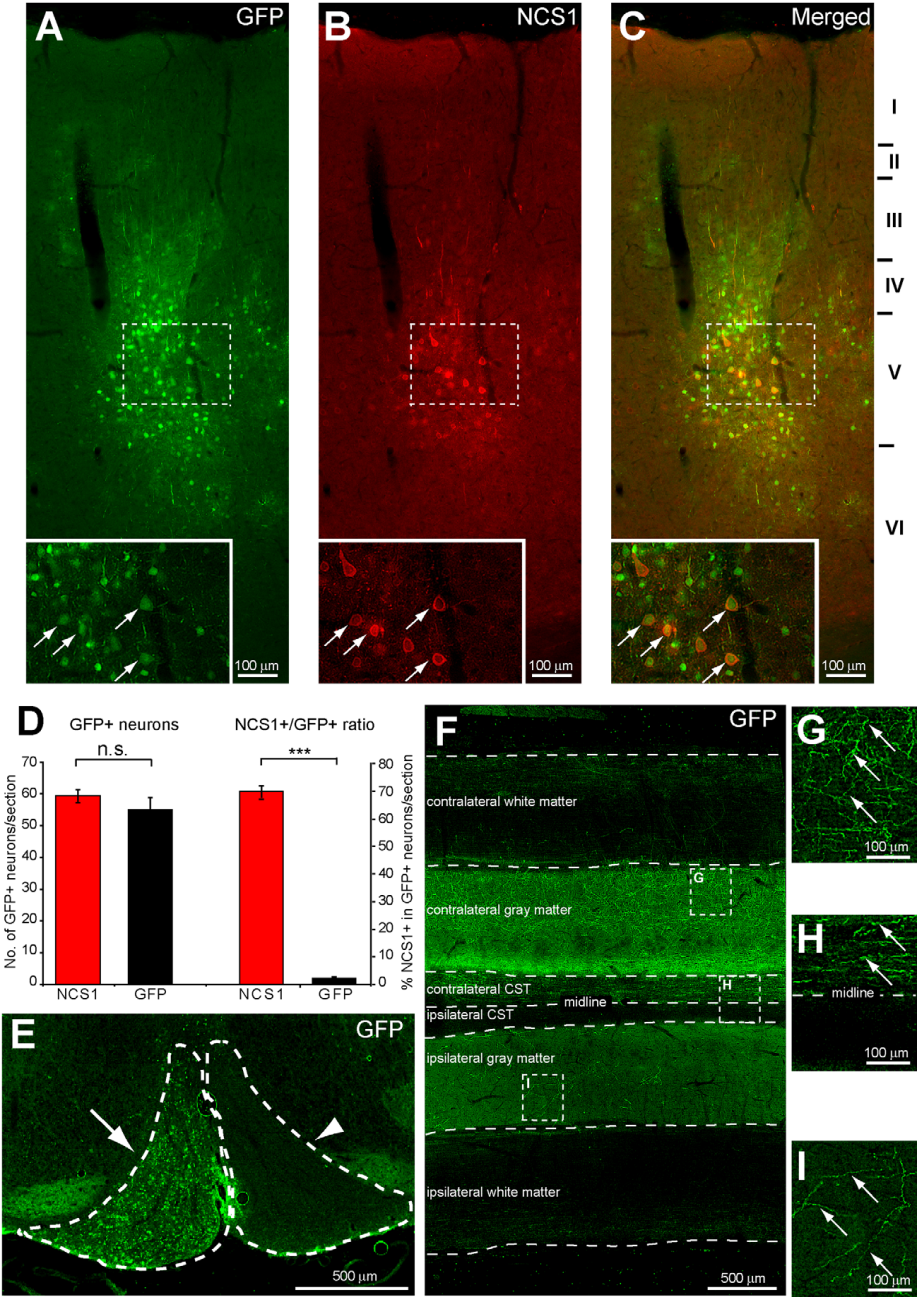
Transduction of corticospinal neurons with HIV-GFP-NCS1 lentivector and labelling of CST axons and collaterals. (A–C) Adult corticospinal neurons coexpressing GFP (green) with overexpresessed NCS1 (red) were observed in the cortical layer V, 3 wk after injection with HIV-GFP-NCS1 lentivector. Insets are higher magnification of dashed boxes in corresponding panels. (D) No significance difference was observed in the number of transduced neurons in layer V of the cortex between the NCS1- and GFP-transduced rats. However, the NCS1-transduced group has significantly more NCS1 positive neurons than the control group. (E) Transverse section showing the pyramidal tract at the medullary level with GFP positive fibers present ipsilaterally (arrow) but not contralaterally (arrowhead) to the left sensorimotor cortex injected with HIV-GFP-NCS1 lentivector. (F) Horizontal section of cervical spinal cord through the base of posterior column showing the corticospinal tract (CST) with extensive GFP-labelled axons and collaterals of an intracortical HIV-GFP-NCS1-transduced rat. Contralateral and ipsilateral sides are in reference to the intracortical injections. (G–I) Higher power images of panel F indicated by dashed boxes showing GFP-labelled collateral sprouts observed in the contralateral gray matter (G) and in the ipsilateral gray matter (I), and GFP-labelled axons in the contralateral CST (H). Data are expressed as mean ± SEM from *n* = 5–6 rats per group with 5–6 sections per animal. *** *p*<0.001, Student's *t* test. Scale bars: (A–C, G–I) 100 µm, (E–F) 500 µm.

With the demonstration that NCS1 overexpression induces neurite sprouting in primary cultured adult cortical neurons, it was next determined whether this also occurred in vivo. HIV-GFP-NCS1 or control HIV-GFP lentivector was injected into the forelimb and hindlimb regions of the left sensorimotor cortex of adult rats. High efficiency was achieved of both GFP and NCS1 expression in the CSN at 3 wk with HIV-GFP-NCS1 (Figure 5A–5C). Similar numbers of GFP labelled neurons in layer V of the sensorimotor cortex were observed in both transduced groups as detected by immunohistochemistry (Figure 5D). Within such GFP labelled neurons, the percentage with NCS1 positive immunostaining was significantly higher in NCS1-transduced neurons than in the control GFP-transduced neurons (Figure 5D). Axons from NCS1-transduced CSN were visible in the pyramidal tract with GFP immunostaining (Figure 5E), in the main dorsal component of the CST, and in its collaterals at the cervical cord level (Figure 5F–5I). These data show that GFP allows a precise identification both of co-labelled transduced neurons overexpressing NCS1 and of their axons, thus obviating the need for later labelling with neuronal tracers or by the use of an independent virus expressing LacZ or GFP.

### NCS1 Overexpression Promotes Uninjured Axon Collateral Sprouting in the Spinal Cord

**Figure 6:**
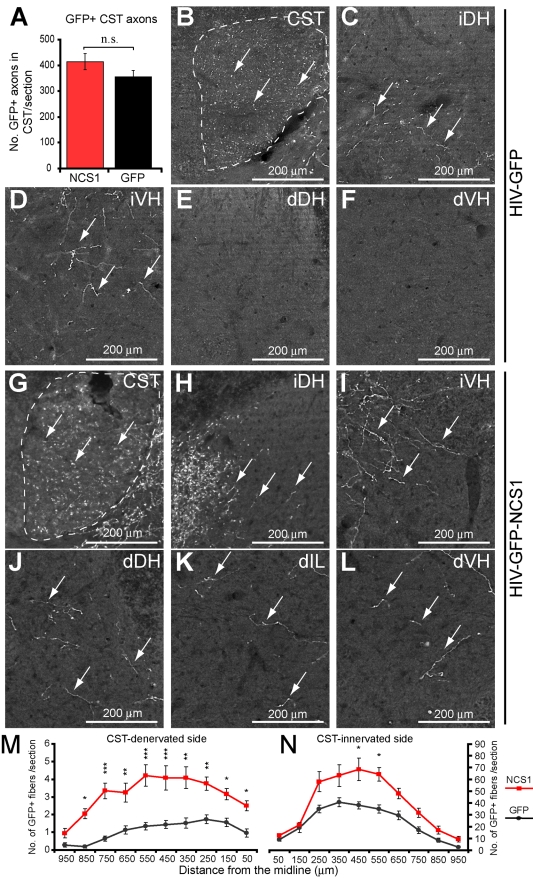
NCS1 overexpression induces cervical axon collateral sprouting from the intact CST after pyramidotomy. (A) No significant difference in number of GFP positive axons in the dorsal corticospinal tract (CST) was observed between the NCS1- (red bar) and GFP- (black bar) transduced groups. (B–F) Photomicrographs of GFP positive fibers in the cervical rat spinal cord following unilateral intracortical injections with control HIV-GFP lentivector and a pyramidotomy. (B–D) On the CST-innervated side of the spinal cord, GFP positive fibers (arrows) were observed in the dorsal CST and dorsal and ventral horns. (E–F) In contrast, at the CST-denervated side of the spinal cord, few GFP positive fibers were detected in either the dorsal or ventral horns. (G–I) In the CST-innervated side of NCS1-transduced rats, GFP positive fibers (arrows) were observed in the dorsal CST and dorsal and ventral horns. (J–L) Furthermore, at the CST-denervated side, GFP positive fibers were now also present in the dorsal and ventral horns and intermediate laminae. (M) Mediolateral spatial distribution of GFP positive fibers throughout the CST-denervated side of the spinal cord with NCS1- (red line) compared to GFP- (black line) transduced rats. The central canal was taken as the midline point indicated as 0 µm. (N) On the CST-innervated side, a discrete region showed a significant difference between the two groups. The complete transverse sections of spinal cord shown in panels B–F and G–L can be seen in Figures S6 and S7, respectively. Dorsal CST (CST); innervated dorsal horn (iDH); innervated ventral horn (iVH); denervated dorsal horn (dDH); denervated intermediate laminae (dIL); denervated ventral horn (dVH). Data are expressed as mean ± SEM from *n* = 5–6 rats per group with 5–6 sections per animal. * *p*<0.05, ** *p*<0.01, *** *p*<0.001, two-way ANOVA, Tukey post hoc test. Scale bar: 200 µm.

**Figure 7:**
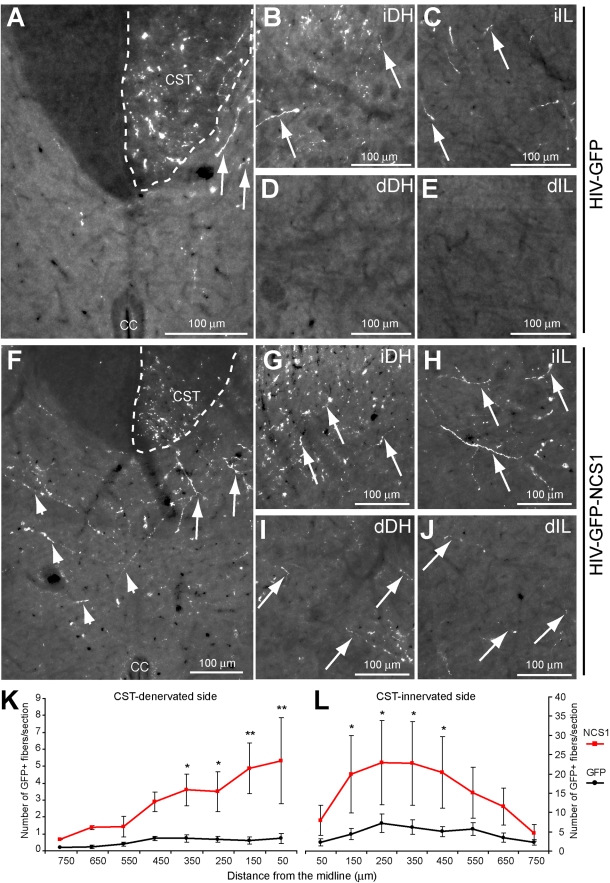
NCS1 overexpression induces lumbar axon collateral sprouting from intact CST after pyramidotomy. Photomicrographs of GFP positive fibers in the lumbar rat spinal cord following unilateral intracortical injections with lentivector and a pyramidotomy. (A–C) In the CST-innervated side of control HIV-GFP-transduced rats, GFP positive fibers (arrows) were observed in the dorsal CST (A), dorsal horn (B), and intermediate laminae (C). (A–E) Few GFP positive fibers were detected crossing the midline into the CST-denervated side demarcated by the central canal (A) or present at the dorsal horn (D) or intermediate laminae (E). (F–H) In the CST-innervated side of NCS1-transduced rats, there were GFP positive fibers (arrows) observed at the dorsal horn (G) and intermediate laminae (H). (F–J) More interestingly, extensive amount of GFP positive fibers were detected crossing the midline into the CST-denervated side (F, arrowheads) and present in the dorsal horn (I) and intermediate laminae (J). (K–L) Mediolateral spatial distribution of GFP positive fibers throughout the CST-denervated and CST-innervated sides of the spinal cord with NCS1-(red line) compared to control GFP- (black line) transduced rats. The central canal was taken as the midline point indicated as 0 µm. Dorsal CST (CST); central canal (cc); innervated dorsal horn (iDH); innervated intermediate laminae (iIL); denervated dorsal horn (dDH); denervated intermediate laminae (dIL). Data are expressed as mean ± SEM from *n* = 5–6 rats per group with 5–6 sections per animal. * *p*<0.05, ** *p*<0.01, two-way ANOVA, Tukey post hoc test. Scale bar: 100 µm.

Adult Wistar rats received unilateral intracortical injections of either HIV-GFP-NCS1 (NCS1-transduced) or the HIV-GFP (control) lentivector 3 wk before receiving on the contralateral side a unilateral pyramidal tract lesion which, in turn, causes CST-denervation of the contralateral side of the spinal cord (Figures S4A and S5A–D). The lesion site was defined with the astrocytic marker GFAP and the loss of PKCγ immunostaining caudal to the lesion site (Figures S5C–D and S8). At 6 wk post-CST-injury, GFP-immunostaining was performed to define axon collateral sprouting from the intact CST at the cervical and lumbar levels and particularly into the CST-denervated side of the spinal cord. The number of GFP-labelled axons in the CST was not significantly different between the control and NCS1-transduced rats (Figure 6A). In control rats, GFP-labelled collaterals were present in the CST-innervated side of the cord but few in the CST-denervated side at the cervical (Figures 6B–6F, 6M–6N, and S6) and lumbar (Figure 7A–7E) levels. In NCS1-transduced rats, GFP-labelled collaterals were present in the CST-innervated side, with a significant increase in the peak number of GFP positive fibres at the mediolateral region (Figures 6G–6I, 6N, 7F–H, 7L, and S7). More importantly, a significant increase also occurred in the number of GFP positive fibers sprouting across the midline into the CST-denervated cord. At the cervical level, GFP positive fibers in the range of 1–2 fibers per section for the control group compared to 4–5 fibers for the NCS1-transduced rats. At the lumbar level, GFP positive fibers of no more than 1 fiber per section for the control group compared to 5–6 fibers for the NCS1-transduced rats. This significant difference was maintained for up to 850 µm and 350 µm from the midline at the cervical and lumbar level, respectively (Figures 6J–6M, 7I–7K, and S7). The completeness of the tract lesions was confirmed by PKCγ immunostaining in the spinal cord (Figure S8). These data show that overexpression of NCS1 in CSN at the cortical level can induce distal axon collateral sprouting across the midline into the CST-denervated side of the cervical and lumbar spinal cord.

### NCS1 Overexpression Promotes Functional Recovery

#### Behavioural studies

**Figure 8:**
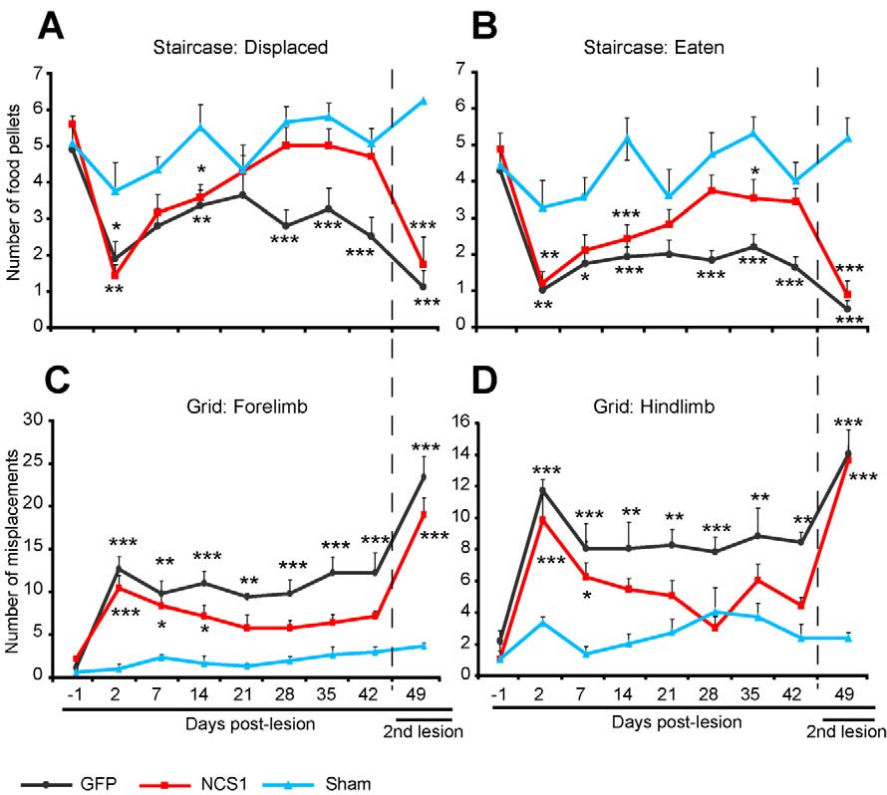
NCS1 overexpression promotes functional recovery. (A–B) In the staircase reaching test, no significant decrease in either the numbers of food pellets displaced or eaten was observed at later time points after treatment with HIV-GFP-NCS1 compared to that of the sham rats. In contrast, significant decrease was observed throughout all time points with the control (HIV-GFP) rats. (C–D) In the grid exploration test, rats injected with HIV-GFP-NCS1 lentivector showed a progressive reduction with time in misplacements of both limbs compared to the sustained lesion-induced increase misplacements in control rats. (A–D) Six weeks after CST-injury and behavioural testing, the remaining intact pyramidal tract was lesioned in a second operation. Rats re-tested 7 d post-operatively exhibited abolishment of the previously observed functional recovery. GFP, black line; NCS1, red line; Sham, blue line. Data are expressed as mean ± SEM from *n* = 8–9 rats per group. * *p*<0.05, ** *p*<0.01, *** *p*<0.001, two-way ANOVA, Tukey post hoc test.

During the 6-wk period after CST-injury, behavioural testing was used to assess sensorimotor function in control (HIV-GFP) and NCS1-transduced (HIV-GFP-NCS1) rats. The sensorimotor function was assessed using the staircase reaching apparatus to test for forelimb extension and grasping movements and the grid exploration test to test for sensory motor function of fore- and hindlimbs. The CST-denervated forelimb of the control and NCS1-transduced groups showed a significant impairment in eaten or food pellet displacement compared to the sham group 2 d post-CST-injury (Figure 8A–8B). However, by 21 d post-CST-injury, the sensorimotor performance of the NCS1-transduced rats greatly improved, with no significant difference from the sham group (Figure 8A–8B). In contrast, forelimb function in the control group remained significantly different from the sham for up to 42 d (Figure 8A–8B). Given that no differences were detected between groups in the CST-innervated forelimb, this indicates that the poor performance of the affected forelimb was due to failure of limb usage rather than the rat's loss of interest in food or locomotion (Figure S9).

In the grid exploration test, a significant difference in limb misplacement was observed in the control group compared with the sham throughout the behavioural testing period (Figure 8C–8D). In contrast, by day 21 post-CST-injury for the forelimb and day 14 for the hindlimb, the NCS1-transduced group showed no significant difference in limb misplacement to the sham group (Figure 8C–8D).

To demonstrate further that overexpressed NCS1 had induced axon collateral sprouting from the intact CST, extending into the CST-denervated side of the spinal cord, a subsequent lesion of the intact pyramidal tract (termed second lesion) was made at medullary level (Figures S4A and S5E–S5H). This second lesion abolished the recovered behaviour in the NCS1-transduced rats and also affected the corresponding lesioned side of the sham group in both the staircase and grid tests (Figures 8A–8D and S9). These data show that overexpression of NCS1 in CSN at the cortical level can induce intact CST to form functional connections in the CST-denervated side of the spinal cord.

#### Electrophysiological studies

**Figure 9:**
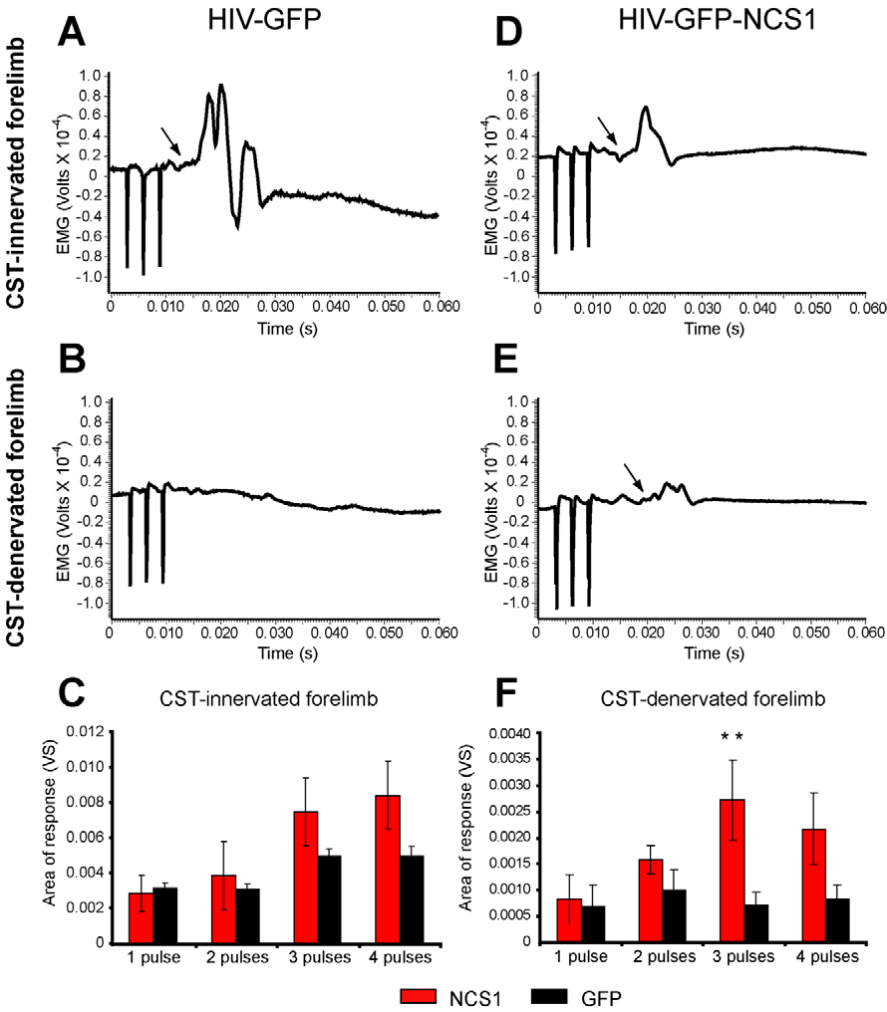
Electromyogram activity observed with NCS1-induced axon collateral sprouting. (A–B) Typical example of electromyogram (EMG) recorded from the CST-innervated forelimb (A) and absent EMG from the CST-denervated forelimb (B) following surface stimulation of the contralateral sensorimotor cortex with 3 pulses (3 ms apart) in rats intracortically injected with control HIV-GFP lentivector. (D–E) In NCS1-transduced rats, an EMG from the CST-innervated forelimb and a delayed EMG from the CST-denervated forelimb following surface stimulation of the contralateral sensorimotor cortex was detected. (C) No significant difference was observed with the CST-innervated forelimb between groups. (F) However, the CST-denervated forelimb shows a significantly greater evoked response after 3 pulses following NCS1-transduced (red bar) compared to the control-transduced (black bar) rats. Data are expressed as mean ± SEM from *n* = 4 rats per group. ** *p*<0.01, two-way ANOVA, Tukey post hoc test.

It is well established in the anesthetised rats that stimulation of the forelimb area of the motor cortex results in contralateral forelimb movements [28] and these persist following ipsilateral pyramidotomy [29]. We have used this approach to assess the physiological status of the intact CST in the two experimental groups. In the present study, electrical stimulation of the transduced motor cortex evoked contralateral movements and EMG responses in the CST-innervated forelimb in both the control and NCS1-transduced groups with 6 wk post-CST-injury (Figure 9A, 9C, and 9D). No significant difference was observed in the EMG responses of the CST-innervated forelimb between the two groups, irrespective of the number of stimulating pulses (Figure 9C). However, in the CST-denervated limb the NCS1-transduced group showed a significantly larger EMG response and elbow flexion after stimulation with three pulses when compared with the control group (Figures 9B, 9E–9F, and S10). To determine if the EMG responses observed in the CST-denervated limb were due to axon collateral sprouting from the intact CST in NCS1-transduced rats, a 1 mm wide chisel was used to section the intact dorsal CST at C4 level. This instantly and completely abolished the elbow flexion and EMG response from the CST-denervated side, and all but abolished that on the CST-innervated side (Figure S10). These data show that the EMG responses on the CST-denervated side were wholly dependent on pathways crossing from the CST-innervated side.

### Delayed NCS1 Overexpression Promotes Axon Sprouting and Regeneration

#### Sprouting of uninjured CST axons

**Figure 10:**
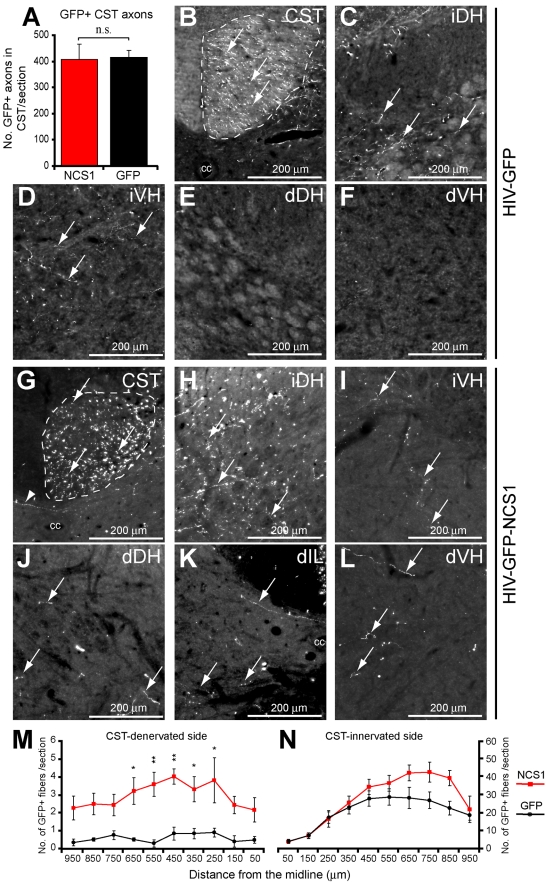
Delayed NCS1 overexpression after pyramidotomy also induces cervical axon collateral sprouting from the intact CST. (A) No significant difference in number of GFP positive axons in the dorsal corticospinal tract (CST) was observed between the NCS1- (red bar) and GFP- (black bar) transduced groups. (B–F) Photomicrographs of GFP positive fibers in the cervical rat spinal cord following a unilateral intracortical injection of control HIV-GFP lentivector 2 d after a pyramidotomy. (B–D) On the CST-innervated side, GFP positive fibers (arrows) are present in the dorsal CST and dorsal and ventral horns. (E–F) However, few GFP positive fibers were detected in the CST-denervated side either at the dorsal or ventral horns. (G–L) Photomicrographs of GFP positive fibers in the cervical spinal cord of NCS1-transduced rats under the same experimental paradigm as the control. (G–I) GFP positive fibers (arrows) were observed in the dorsal CST and dorsal and ventral horns. (J–L) More interestingly, GFP positive fibers were also present in the CST-denervated side of the dorsal and ventral horns and intermediate laminae. (M–N) Mediolateral spatial distribution of GFP positive fibers throughout the CST-denervated and CST-innervated sides of the spinal cord with NCS1- (red line) compared to GFP- (black line) transduced rats. The central canal was taken as the midline point indicated as 0 µm. Dorsal CST (CST); central canal (cc); innervated dorsal horn (iDH); innervated ventral horn (iVH); denervated dorsal horn (dDH); denervated intermediate laminae (dIL); denervated ventral horn (dVH). Data are expressed as mean ± SEM from *n* = 4–5 rats per group with at least 6 sections per animal. * *p*<0.05, ** *p*<0.01, two-way ANOVA, Tukey post hoc test. Scale bar: 200 µm.

To investigate whether a delayed NCS1 overexpression in CSN can also promote axon collateral sprouting to the same extent as priming the CSN for 3 wk, adult Wistar rats received unilateral intracortical injections of either HIV-GFP-NCS1 or the control HIV-GFP lentivector 2 d after receiving on the contralateral side a unilateral pyramidal tract lesion (Figure S4B). At 4 wk post-CST-injury, the number of GFP positive axons at the CST was not significantly different between the groups (Figure 10A–10B and 10G). There were more GFP positive collaterals present in the CST-innervated side of the NCS1-transduced rat cervical spinal cord than the control GFP-transduced rats, but was not significant (Figure 10C–10D, 10H–10I, and 10N). More importantly, a significant increase in the number of GFP positive collaterals was detected sprouting across the midline into the CST-denervated side of the NCS1-transduced rat spinal cord compared to the control GFP-transduced rats (Figure 10E–10F and 10J–10M).

These data show that delaying the overexpression of NCS1 in CSN after injury can still induce intact CST axons to sprout into the CST-denervated side of the spinal cord.

#### Sprouting and regeneration of injured CST axons

**Figure 11:**
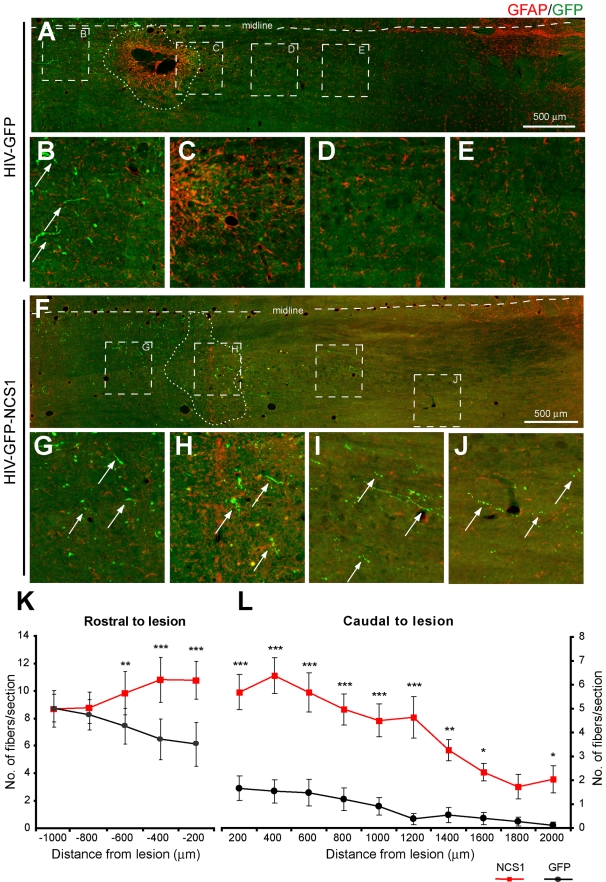
Delayed NCS1 overexpression after pyramidotomy induces axonal sprouting and regeneration from the lesioned CST. Photomicrographs of GFP positive fibers in horizontal sections of the caudal medulla oblongata following a unilateral intracortical injection of lentivector 2 d after a pyramidotomy. Images are orientated in the rostral-caudal orientation from left to right. GFP positive fibers are depicted as green and the reactive astrocytes demarcating the lesion site are depicted as red. (A–E) In control HIV-GFP-transduced rats, GFP positive fibers (arrows) are seen approaching the rostral edge of the lesion (B) but few GFP positive fibers distal to the lesion. (F–J) In HIV-GFP-NCS1-transduced rats, GFP positive fibers (arrows) are seen approaching the lesion site from the rostral side. Equally important, GFP positive fibers (arrows) are present caudally with a distance of up to 2 mm distal to the lesion. (K–L) Quantification of GFP labelled fibers in the side ipsilateral to the pyramidal tract lesion demarcated by the midline with the lesion site as 0 µm. The HIV-GFP-NCS1-transduced rats showed a significant increase in the number of GFP positive fibers along the rostral and caudal regions to the lesion when compared with the control HIV-GFP-transduced rats. Panels B–E and G–J are higher magnifications of areas indicated by dashed boxes of panels A and F, respectively. Data are expressed as mean ± SEM from *n* = 4–5 rats per group with 5–6 sections per animal. * *p*<0.05, ** *p*<0.01, *** *p*<0.001, two-way ANOVA, Tukey post hoc test. Scale bar: 500 µm.

To investigate whether a delayed NCS1 overexpression of axotomized CSN can promote axonal sprouting and regeneration, adult Wistar rats received unilateral intracortical injections of either HIV-GFP-NCS1 or the control HIV-GFP lentivector 2 d after receiving on the ipsilateral side a unilateral pyramidal tract lesion at the medullary level, approximately 2 mm rostral to the decussation (Figures S4C and 5A–5B). At 4 wk post-CST-injury, the number of GFP positive axons distal rostral to the lesion site was similar between the control GFP- and NCS1-transduced rats (Figure 11A–11B, 11F, 11G, and 11K). However, at close proximity to the lesion site, both rostrally and caudally, NCS1-transduced rats have a significantly greater number of GFP positive fibers than the control group suggesting axonal sprouting (Figure 11A–11C, 11F–11H, and 11K–11L). This significant difference in number of GFP positive fibers was detected caudally for up to 2 mm from the lesion site, suggesting regeneration of the lesioned CST axons, as local axonal sprouting rarely exceeds 1 mm from the lesion site [30]. At their maximum, NCS1-transduced rats had 11 fibers per section compared to 3 fibers per section in the control GFP-transduced rats (Figure 11I-11L).

These data show that delaying the overexpression of NCS1 in axotomized CSN can induce axonal sprouting and axonal regeneration.

### NCS1 Overexpression Promotes Neuroprotection in Axotomized CSN

**Figure 12:**
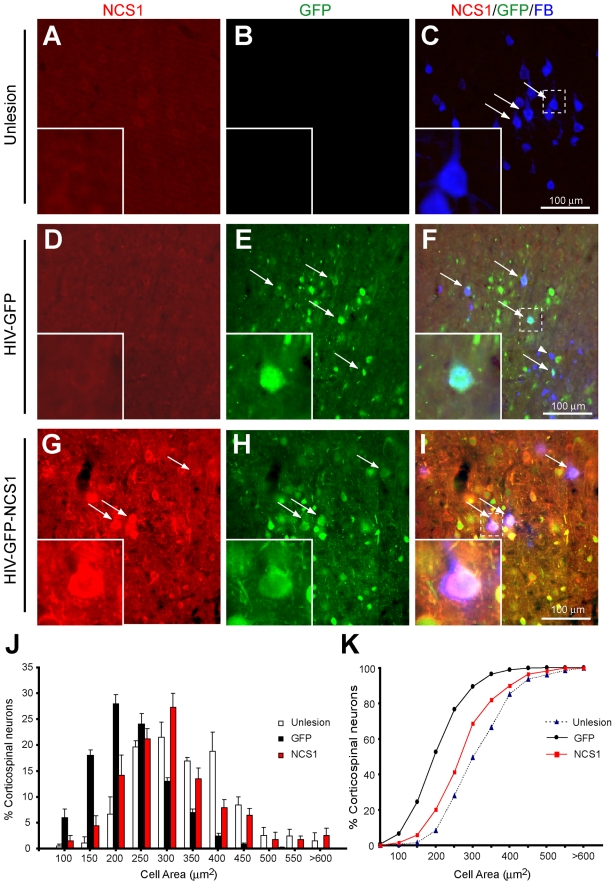
NCS1 overexpression induces neuroprotection in axotomized corticospinal neurons after pyramidotomy. Photomicrographs of corticospinal neurons (CSN) in coronal sections of the sensorimotor cortex following a unilateral intracortical injection of lentivector and a pyramidotomy. Fast Blue retrograde tracer was injected into the lesion site to enable identification of CSN at the sensorimotor cortex. In unlesioned rats, Fast Blue was injected into the pyramidal tract carefully to minimise any lesioning effect. Cortical sections were immunostained for NCS1 (red) and GFP (green). The Fast Blue tracer was visible without the need of immunostaining (blue). (A–C) In unlesioned rats, CSN with large healthy somata (arrows) can be observed with low NCS1 and no GFP immunostaining. (D–F) In contrast, the control HIV-GFP-transduced rats with a pyramidotomy have CSN with severely shrunken somata with GFP (arrows) or without GFP (arrowhead) labelling. (G–I) In HIV-GFP-NCS1-transduced rats with a pyramidotomy, GFP-labelled CSN with elevated NCS1 expression have similar somata size as the unlesioned rats (arrows). (J–K) Cell size distributions of CSN determined from colabelled Fast Blue and GFP (Fast Blue alone for unlesioned rats) neurons. The CSN from control HIV-GFP- (black bar and line) but not HIV-GFP-NCS1- (red bar and line) transduced rats had undergone significant atrophy, causing a leftward shift in the cell size distribution towards the small cell areas compared to the CSN from unlesioned rats (white bar and dotted line). Statistical significance of *p*<0.05 was obtained using the Kolmogorov-Smirnov test.

It has been shown that a pyramidal tract lesion in adult hamster causes CSN to become atrophied after 2 wk post-injury [31]. To investigate whether NCS1 overexpression can prevent adult axotomized CSN from atrophy, adult Wistar rats received unilateral intracortical injections of either HIV-GFP-NCS1 or the control HIV-GFP lentivector 1 wk before an ipsilateral pyramidal tract lesion at the medullary level. To identify CSN, the retrograde Fast Blue tracer was injected directly into the lesion site immediately after sectioning (Figure S4D). Controls were unlesioned rats with Fast blue injected into the pyramidal tract at the medullary level with no intracortical lentiviral injection. After 2 wk post-lesion, the axotomized CSN in control GFP-transduced rats that have low NCS1 levels exhibited significant cell soma shrinkage compared to the large and healthy CSN in unlesioned rats (Figure 12A–12F and 12J–12K). In contrast, the axotomized CSN in NCS1-transduced rats that have high NCS1 levels did not exhibit significant cell soma shrinkage compared to the CSN in unlesioned rats (Figure 12G–12K).

These data suggest NCS1 overexpression in CSN exerts a neuroprotective effect on axotomized CSN.

## Discussion

This present study demonstrates that the intracellular levels of NCS1 in adult cortical neurons can be significantly elevated by transduction with a lentiviral vector. In culture, neurons overexpressing NCS1 develop extensive neurite sprouting which by immunocytochemistry and Western blotting was shown to be via Akt phosphorylation. Similarly, analogous experiments conducted in vivo show that CSN overexpressing NCS1 with intact CST axons can undergo distal collateral sprouting and cross the midline into the CST-denervated side of the spinal cord. This anatomical plasticity is also functional as demonstrated by the behavioural and electrophysiological outcomes in NCS1-transduced adult rats. Furthermore, studies on the axotomized CSN show that NCS1 overexpression not only induces axonal sprouting and regeneration at the lesion site but also exerts a neuroprotective affect on injured CSN.

To date, several therapies for spinal cord injury models have shown both significant axonal regeneration [6]–[9] and anatomical plasticity [10]–[14] within the spinal cord. However, the intracellular mechanisms for these therapies have been little investigated. Only the purine-sensitive ste20-like protein kinase (Mst3b) has been linked to the anatomical plasticity observed with the purine nucleoside inosine [11],[26]. Interestingly, Mst3b has been shown selectively to induce outgrowth only from axons and not dendrites [26]. Conversely, in the present study, NCS1 overexpression induces sprouting from both axons and dendrites in cultured neurons, suggesting the growth induction process of NCS1 is non-selective. However, despite the existence of morphologically and molecularly distinct differences between dendrites and axons, neurons have been shown to have the capacity to generate axons from dendrites [32].

In addition to observing neurite sprouting in vitro, we demonstrated an NCS1 mediated axon collateral sprouting in vivo following unilateral pyramidotomy. The HIV-GFP-NCS1 lentivector injected into the cortex enabled GFP labelling of neurons overexpressing NCS1. The GFP labelling allowed visualisation and quantification of sprouting of the CST axons without the need of applying an independent tracer, and such labelling of axons and their collaterals can be detected as far distal as the lumbar region.

We report for cervical region the number of GFP-positive collateral fibers from uninjured CST axons that have crossed the midline into the CST-denervated side of the spinal cord, as measured per 40 µm section, is 1–2 for the control and 4–5 for the NCS1-transduced rats. It is of interest to consider the possible total number of crossing fibers over the relevant cervical region. The length of the adult rat C5–C8 cervical cord containing the majority of forelimb motoneurons is approximately 11 mm [33],[34]. Extrapolating using these data, the NCS1-transduced rats would have approximately over 800 additional collaterals to account for the functional plasticity demonstrated behaviourally and electrophysiologically in these rats. Furthermore, this recovery was shown to be dependent on the collaterals provided by the intact CST as demonstrated by its loss following the second pyramidotomy as well as the loss of the crossed EMG activity when the intact CST axons were sectioned in the terminal experiment.

We have previously induced axonal regeneration by lentivector overexpression of retinoic acid receptor β2 (RARβ2) [15],[16]. In this study, NCS1 overexpression has been demonstrated in axotomized CSN to induce axonal sprouting and regeneration. Recently, we showed that the retinoic acid receptor beta agonist (CD2019) overcomes inhibition of axonal outgrowth via the PI3/Akt pathway in injured adult rat spinal cord [35]. From our unpublished data, lentivector overexpression of RARβ2 also induces an upregulation of NCS1 as initially detected by microarray analysis and confirmed immunohistochemically and with real-time PCR. Thus this present study suggests that upregulation of NCS1 is a major intracellular target linking RARβ2 to the PI3K/Akt pathway in inducing anatomical plasticity and axonal regeneration. A similar explanation may account for GDNF-induced anatomical plasticity as other studies have shown NCS1 is upregulated by GDNF [14],[19],[36].

The successful regenerative responses of CSN after pyramidotomy with delayed post-injury NCS1 overexpression suggests axonal sprouting and regeneration can occur without the need to prime with overexpression of NCS1. These data provide promising support for NCS1 overexpression as a possible therapeutic treatment for CNS injury in a clinical setting.

This study also reveals a neuroprotective feature of NCS1 overexpression in reducing cell shrinkage due to retrograde effects of axotomy. Others have shown that neurotrophic factors can prevent atrophy or death of axotomized CSN [31],[37]. Recently, Chondroitinase ABC, which is known to remove the inhibitory scarring at the injury site, has been shown to induce neuroprotection of CSN via a possible retrograde effect mediated at the injured mouse spinal cord [38]. However, our study demonstrates that NCS1 overexpression can increase the intrinsic capacity of CSN to overcome the inhibitory environment and even compensate for the apparent lack of trophic support associated with CNS injury.

Our study establishes that NCS1 is an important intracellular component in the regulation of axonal sprouting and regeneration, and neuroprotection in central neurons of an adult mammalian nervous system, as recently shown for the peripheral nervous system in chick embryo studies on dorsal root ganglion (DRG) neurons [24]. Furthermore, the PI3K/Akt pathway mediating these responses in vitro and in vivo is consistent with the experiments on primary cultured adult DRGs and perinatal cortical neurons linking Akt activation with neurite outgrowth [39],[40] and the survival of primary cultured embryonic cortical neurons [19]. The opposite conclusion, that NCS1 contributes to a retardation of neurite growth, may relate to the use of a rat adrenal medullary pheochromocytoma cell line (PC12 cells) and the additional need for NGF to promote differentiation into sympathetic neuron-like cells [41],[42].

In summary, the limited ability of adult CST neurons to undergo functional sprouting may be due to low endogenous levels of NCS1. Thus NCS1 emerges as a potential intracellular target for therapeutic intervention following injury to the central nervous system.

## Materials and Methods

### Minimal Lentiviral Vector Construction and Production

The complete cDNA sequence of the rat NCS1 was generated by PCR from adult rat cortex using the following forward primer; 5′-ATGGGGAAATCCAACAGCAAG-3′; and the reverse primer, 5′-CTATACCAGCCCGTCGTAGAG-3′ then cloned into a pCR2.1-TOPO vector (Invitrogen). The *ncs1* gene was inserted under the control of a CMV promoter in a human immunodeficiency virus type 1 (HIV1) vector containing a 5′ central polypurine tract (cPPT) element and a 3′ woodchuck post-transcriptional regulatory element (WPRE) enhancer. To allow for coexpression of NCS1 and the enhanced green fluorescent protein (GFP), the *eGFP* gene was inserted into the *Cla*1 site under the control of a SFFV promoter. Viral vector stocks, pseudotyped with the VSV-G envelope glycoprotein, were prepared by triple plasmid transient transfection of HEK293T cells as previously described [43]. The titre of pRRL-CMV-NCS1-SFFV-eGFP (for simplicity termed HIV-GFP-NCS1) was 3.3–4.4×10^8^ TU ml^−1^ and pRRL-CMV-eGFP (for simplicity termed HIV-GFP) was 4.7–4.8×10^8^ TU ml^−1^ determined by transient transfection of the HEK 293T cell line and analysed by flow cytometry.

### Primary Adult Cortical Neuron Cultures

Adult cortical neurons were cultured as previously described [16],[44]. Adult male Wistar rats (220–250 g) were overdosed with sodium pentobarbitone (Lethobarb), transcardially perfused with heparinized saline, and the cortices removed with as little white matter as possible. The cortices were cut into 0.5 mm longitudinal sections using a McIlwain tissue chopper before digestion in 2 mg/ml papain at 30°C for 30 min. Cortical neurons were mechanically dissociated with a glass Pasteur pipette and separated from debris by centrifugation in four 1 ml steps of Optiprep in HibernateA/B27 medium (7.5%, 10%, 12.5%, and 17.5%) at 600 g for 15 min. Fractions containing neurons were collected, washed, and resuspended in NeurobasalA/B27 medium for plating at a density of 3,000 cells per well on poly-D-lysine (10 µg/ml) pre-coated cover slips. The neurons were allowed to settle onto the cover slips for 1 h, and after washing, HIV-GFP-NCS1 or control HIV-GFP lentivector was added to the media at MOI 10. The neurons were left for a further 3 days in vitro (DIV) before immunocytochemical processing.

For phospho-Akt studies, the reversible PI3K/Akt inhibitor LY294002 (hydrochloride, 40 µM dissolved in DMSO, Tocris Biosciences) or DMSO alone at a final concentration of 0.01% was added to the cultures at 1 DIV and 2 DIV. The neurons were harvested at 3 DIV.

After 3 d in culture, neurons were fixed with 4% paraformaldehyde for 20 min and then permeablized with cold methanol for 3 min. The neurons were washed three times for 5 min with 0.01 M phosphate-buffered saline (PBS) before 2 h incubation with chicken anti-GFP (1∶1000, ab13970, Abcam), rabbit anti-NCS1 (1∶500, NL3750, BioMol International), rabbit anti-phospho-Akt (1∶200, Cell Signalling Technology), or rabbit anti-MAP2 (1∶1000, AB5622, Chemicon). The cover slips were washed 3×5 min with PBS and then incubated with donkey anti-rabbit Alexa Fluor 546 and goat anti-chicken Alexa Fluor 488 secondary antibodies (1∶2000, Molecular Probes) for 45 min. After 3×5 min PBS washes, they were mounted with FluorSave reagent containing 0.5 µl DAPI (10 µg/ml) to visualise cell nuclei. The staining with phalloidin-TRITC (1∶100, P5282, Sigma) was carried out 1 h before immunostaining with other antibodies was carried out.

### Image Analysis and Quantification

Image analysis and quantification was made with the observer blinded to the group assignment as previously described [16],[45],[46]. Analyses were restricted only to transduced neurons immunoexpressing GFP. For each experimental group, 50–100 GFP-positive neurons were captured randomly using a Zeiss Axioplan 2 fluorescence microscope. The soma of each neuron was outlined to obtain the fluorescent intensity using the Axiovision V4.6 software to determine the neuronal levels of NCS1 and phospho-Akt immunoreactivity. To minimise variability between each image, the capture settings were fixed throughout the whole study. The number of neurite sprouts from the cell bodies and of the longest neurites, of length greater than cell body diameter was determined. To differentiate whether a GFP positive neurite was a dendrite or an axon, the specific dendritic marker microtubule-associated protein 2 (MAP2) was used. Neurites with strong and weak MAP2 immunostaining were identified as dendrites and axons, respectively.

### Western Blot Analysis

Western blots were carried out as previously described [47],[48]. After 3 d in culture, primary adult cortical neurons transduced with either HIV-GFP-NCS1 or the control HIV-GFP vector, with or without the PI3K/Akt inhibitor LY294002, media were removed and neurons were harvested in 250 µl ice-cold lysis buffer (20 mM HEPES pH 7.4, 100 nM NaCl, 100 mM NaF, 1 mM Na_3_VO_4_, 5 mM EDTA, 1% Nonidet P-40 and 1× protease inhibitor cocktail; Roche). To obtain sufficient protein, the same 250 µl lysis buffer was used in three cultured wells and the lysates rotated for 2 h at 4°C. After centrifugation at 13,500 g for 15 min at 4°C, the supernatant was collected and total protein concentration was determined using a bicinchoninic acid protein assay kit (Pierce).

Intracortical injections of either HIV-GFP-NCS1 or HIV-GFP lentivector in adult male Wistar rats (*n* = 4–5 per group) were carried out as described below. To determine the role of Akt activation, the PI3K/Akt inhibitor LY294002 (100 mM) or vehicle (DMSO) was injected intracerebroventricularly via an externalised catheter on every other day of the third post-injection week. At the end of the third week, rats were sacrificed and the injected region of the cortex was freshly and quickly removed and stored at −80°C until further processed. The protein obtained for Western blotting was extracted as described above.

Fifteen micrograms of total protein were electrophoresed on 12% acrylamide gel before transfer onto Hybond P membranes (Amersham) and incubated overnight at 4°C with rabbit anti-phospho-Akt (Ser 473, 1∶100, #3787S, Cell Signalling Technology), rabbit anti-NCS1 (1∶1000, NL3750, BioMol International), or mouse anti-β III tubulin (1∶1000, G712A, Promega). Visualisation was performed using secondary antibodies, donkey anti-rabbit IRDye-800CW, and goat anti-mouse IRdye-680CW (LI-COR Biosciences). Fluorescent blots were imaged on the Odyssey Infrared Imaging System (LI-COR Biosciences). To allow for visualisation of the total Akt on the same blot as phospho-Akt (both antibodies were raised in the same species), the blot was first stripped with buffer (62.5 mM Tris-HCl pH 6.8, 2% SDS, 100 mM *β*-mercaptoethanol) before re-blotting with rabbit anti-Akt (1∶100, #9272, Cell Signalling Technology). Western blotting was carried out with 3–5 independent samples.

### Viral Vector Delivery to the Sensorimotor Cortex

The surgery was performed aseptically in accordance with UK Home Office regulations as previously described [16]. Briefly, adult male Wistar rats (*n* = 8–9 per group) were anaesthetized using a combination of ketamine and medetomidine, then fixed in a stereotaxic frame. The skull was exposed and injections were made at a depth of 2 mm dorsoventrally into the sensorimotor cortex region using the injection coordinates as determined from a microstimulation mapping study [28]. These were, with reference to bregma (AP, anterior-posterior; L, lateral); AP: −1.5 mm, L: 2.5 mm; AP: −0.5 mm, L: 3.5 mm; AP: +0.5 mm, L: 3.5 mm; AP: +1.0 mm, L: 1.5 mm; AP: +1.5 mm, L: 2.5 mm; AP: +2.0 mm, L: 3.5 mm. At each site, 1 µl of HIV-GFP-NCS1 or control HIV-GFP lentivector was directly injected at a rate of 0.2 µl/min using a microinfusion pump via a finely pulled glass micropipette and left in situ for a further 1 min. HIV vector pseudotyped with a VSV-G envelope produced strong expression and anterograde labelling [49]. Three weeks after viral injection, a unilateral pyramidal tract lesion at the level of medulla was performed as described previously [50]. A ventral midline incision was made and the occipital bone exposed by blunt dissection. The ventrocaudal part of the bone was partially removed using fine rongeurs, exposing the right medullary pyramid. The dura was opened and the right pyramidal tract was sectioned approximately 2 mm rostral to the decussation with fine iridectomy scissors using the basilar artery as the midline. Sham operated rats received similar surgery without incision of the tract. In another group of experiments, the left intact pyramidal tract was transected in a second operation.

In the delayed lentivector transduction studies, adult Wistar rats (*n* = 5–6 per group) received intracortical lentiviral injections 2 d after a unilateral pyramidal tract lesion as described above. To study the sprouting effect of delayed lentivector transduction on uninjured and injured CST axons, the lentiviral injections were administered into the sensorimotor cortex corresponding to the unlesioned and lesioned pyramidal tract, respectively. After 4 wk post-surgery, the rats were perfused transcardially with 4% paraformaldehyde and tissue collected for histology.

In the neuroprotection study, adult Wistar rats (*n* = 4–5 per group) received intracortical lentiviral injections 1 wk before receiving on the ipsilateral side a unilateral pyramidal tract lesion as described above. Using a microinfusion pump, Fast Blue tracer (200 nl, 2% wt/vol PBS, EMS-Chemie GmbH) was administered at a rate of 0.2 µl/min into the lesion site via a finely pulled glass micropipette and left in situ for a further 1 min. Unlesioned rats without intracortical lentiviral injection had Fast Blue tracer (2%, 200 nl) injected into the pyramidal tract at the medullary region. Care was taken to minimise axonal damage by the injection process. After 2 wk post-injection of tracer, the rats were perfused transcardially with 4% paraformaldehyde and tissue collected for histology.

### Histological Assessment of Vector Transduction and Axon Collateral Sprouting

#### Immunohistochemical staining of the spinal cord

Axon collateral sprouting of the intact CST across the midline into the CST-denervated side of the cord was assessed by staining for the GFP immunopositive fibers. Forty µm transverse sections of the cervical (C5–C8 level) and lumbar (L3–L6 level) spinal cord were cut using a vibratome. Sections were collected in cold 0.01 M PBS with 0.1% sodium azide with the format of one section in each of 32 wells and this was repeated so each well had at least six sections containing the corresponding spinal cord levels. Sections chosen from randomly selected wells were rinsed in 3×5 min PBS-T (0.01 M PBS with 0.3% Triton X-100), then incubated with 0.3% H_2_0_2_ for 30 min. After a further 3×5 min washes with PBS-T, the sections were incubated overnight at room temperature with rabbit anti-GFP (1∶2000, A11122, Invitrogen). The next day, the sections were washed in 3×5 min PBS-T before incubation with goat anti-rabbit biotin (1∶400, BA-1000, Vector Laboratories) for 1 h 30 min. After 3×5 min PBS-T washes, sections were incubated in avidin-biotin-peroxidase complex (Vectastain ABC Elite Kit, Vector Laboratories) for 30 min at room temperature. Following 3×5 min washes in PBS-T, sections were incubated in tyramide (1∶75, NEN Life Sciences) for 10 min. After a further 3×5 min wash in PBS-T, the sections were incubated with extra-avidin FITC (1∶400, E2761, Sigma) for 2 h before the sections were washed, mounted, and cover slipped. For quantitative analysis, five to six sections per animal (experimenter was blind to group assignment) stained for GFP were selected and captured as a complete spinal cord image at 10× magnification with five *z*-axis planes (as not all fibers were in the same plane) using a Zeiss Axioplan 2 microscope and Axiovision V4.6 program. The number of GFP positive fibers in the CST was taken as the number of stained axons. The number of CST axon collaterals in the gray matter of the CST-innervated and -denervated side was assessed by counting the number of fibers crossing a virtual line at each 100 µm interval lateral from the midline.

The immunostaining for PKCγ in the spinal cord involved washing the sections in 3×5 min PBS-T before overnight incubation at room temperature with rabbit anti-PKCγ (1∶500, SC-211, Santa Cruz). The next day, sections were washed with 3×5 min PBS-T, then incubated with goat anti-rabbit Alexa Fluor 488 (1∶1000, A11035, Molecular Probes) for 3 h. Following a 3×5 min PBS-T wash, sections were mounted and cover slipped.

#### Immunohistochemical staining of the brainstem

Axon collateral sprouting of the injured CST across the lesion site at the caudal medullary level was assessed by staining for the GFP positive fibers. Forty µm horizontal sections of the caudal medulla oblongata including the lesion site were cut using a vibratome. The immunostaining for GFP positive fibers in the brainstem was carried out with the tyramide signal amplification procedure as mentioned above. After the sections were immunostained for GFP, they were then incubated overnight with rabbit anti-GFAP antibody (1∶500, Z0334, Dako) to further highlight the lesion site with immunostained reactive astrocytes. The next day, sections were washed 3×5 min in PBS and then incubated in donkey anti-rabbit Alexa Fluor 568 (1∶1000, Molecular Probes) for 3 h at room temperature. Following a further 3×5 min wash in PBS, sections were mounted and cover slipped.

For quantitative analysis, five to six horizontal sections per animal at the level of the pyramidal tract lesion stained for GFP and GFAP were selected (experimenter was blind to group assignment) and captured as a complete horizontal section of the medulla oblongata at 10× magnification with four *z*-axis planes (as not all fibers were in the same plane) using a Zeiss Axioplan 2 microscope and Axiovision V4.6 program. The number of CST axon collaterals was assessed by counting the number of fibers crossing a virtual line at each 200 µm interval rostral and caudal to the lesion site and within the ipsilateral side demarcated by the reactive astrocytes and midline, respectively.

#### Immunohistochemical staining of the cortex

The expression of NCS1 and GFP in the cortex of whole rats was assessed by immunohistochemistry. Forty µm coronal sections of the cortex between bregma −2.5 mm and +3.0 mm were cut using a vibratome. Sections were collected and processed as described above. To visualise NCS1 using rabbit anti-NCS1 (1∶500, BioMol International) in histological sections, an antigen retrieval step was required using the antigen unmasking solution (H-3300, Vector Labs) according to manufacturer's instructions. Chicken anti-GFP (1∶1000, ab13970, Abcam) was used to detect GFP positive neurons. Standard immunohistochemical procedures were followed with donkey anti-rabbit Alexa Fluor 568 and goat anti-chicken Alexa Fluor 488 secondary antibodies. Dual or triple colour images of the layer V cortical region were captured at 20× magnification and analysed using the AxioVision V4.6 program. At least six transduced sections were analysed and quantified per animal (*n* = 4–7 per group).

### Cell area Analysis of CSN with Fast Blue Labelling

The analysis of atrophy in CSN was carried out as previously described [38]. The cell area of CSN co-labelled with Fast Blue tracer and GFP immunostaining were acquired using the AxioVision V4.6 program by an investigator blinded to the treatment groups. In unlesioned rats, Fast Blue traced CSN from similar coronal levels as for the lentivector transduced rats were analyzed. Size and frequency distributions of CSN were determined for each rat and a mean distribution calculated for each treatment group. At least six transduced sections were analysed and quantified per rat (*n* = 4–5 per group). A total of over 2,400 neurons were analyzed.

### Behavioural Assessment

Following unilateral pyramidotomy, functional recovery was assessed behaviourally using the staircase reaching and grid exploration tests at 2 d post-lesion and then weekly for 6 wk as described previously [15],[16],[50]. In the staircase reaching test, the rats were trained to reach and grasp the food pellets from a baited double staircase (Campden Instruments) before CST lesion. This test allows assessment of extension and grasping ability independently for each forelimb. On the testing day, rats were placed in the staircase box for 15 min and the number of food pellets removed or displaced was recorded. In the grid exploration test, the rats were allowed to explore the grid freely (40 cm×60 cm containing 5 cm×5 cm mesh, raised 50 cm high) where at least 50 forelimb and 20 hindlimb steps were recorded, typically made within 3 min. The “free” exploration removes any possible learning effect due to training as no pre-training was required and that the rats never move around the grid in the same pattern. The grid exploration captured on video camera was replayed and analysed for limb misplacement on the grid. Analysis involved counting the number of limb misplacement from the first 30 forelimb and 20 hindlimb placements of each rat, to ensure no bias between animals and groups. At the end of the behavioural assessment at 6 wk, the rats were sacrificed and perfused transcardially with 4% paraformaldehyde and tissue collected for histology.

### Electrophysiological Assessment

Control rats (*n* = 4) received intracortical injections of HIV-GFP while NCS1-transduced rats (*n* = 4) received HIV-GFP-NCS1 followed by a unilateral pyramidotomy on the right side 3 wk later as described above. All electrophysiological measurements were performed at least 6 wk post-injury under urethane (1.25 g/kg body weight, i.p.) anesthesia. Following tracheotomy, the rat was fixed into a frame by ear bars and spinal clamps such that the forelimbs were fully pendent. A pair of hooked stainless steel wires insulated to the tip was inserted into the tricep brachii of both forelimbs approximately 6 mm apart for EMG recording. The area of the sensorimotor cortex where lentiviral vectors were injected was exposed by craniotomy, covered by mineral oil, and stimulated through a flat ended silver wire electrode (0.5 mm diameter) ensheathed with plastic to its tip to minimize surface spread of the stimulating current. A 2 mm diameter anode was placed on the skull periosteum rostral to the stimulating electrode. The stimuli repeated at 1 Hz consisted of 1 to 4 pulses, 3 ms apart, and 0.1 ms duration from an isolated stimulator (Digitimer DS2A). The final stimulation site was selected after systematic mapping with varying stimulus parameters until a discrete contralateral (left) forelimb movement was observed with a clear EMG response and a threshold below 25 V for the least number of effective pulses. The EMG was amplified (LF, 30 ms TC; HF, 3 KHz) and digitized using a CED 1401 interface with a sampling rate of 10 kHz. The area of the EMG response (Vs) was measured from 20 averaged responses using Spike 2 V5.0 software.

To check that the EMG response from the CST-denervated forelimb was dependent on contralateral CST input to the spinal cord, the right dorsal CST was transected at the cervical C4 level using a chisel formed by flattening a G25 needle.

### Statistical Analysis

Data were analyzed using SigmaStat 3.5 software. Reported values are expressed as mean ± SEM. The in vitro experiments, Western blot analysis, and number of GFP positive axons in the dorsal CST were analyzed with Student's *t* test. The GFP immunopositive axon collaterals and sprouts in the cord and brainstem, behavioural tasks, and electrophysiology were analyzed with two-way ANOVA followed by Tukey's post hoc test. The cell size cumulative frequency distribution of CSN was analyzed with a two-sample Kolmogorov-Smirnov test, performed against a significant threshold of 0.05 to correct for multiple testing.

## Supporting Information

Figure S1 **Further examples of NCS1 overexpression promoting neurite sprouting in vitro.** (A–F) Primary cultured adult cortical neurons transduced with control HIV-GFP lentivector showing low levels of NCS1 immunostaining and limited neurite sprouts after 3 d in vitro. (G–L) Neurons transduced with HIV-GFP-NCS1 have higher levels of NCS1 immunostaining and a greater number of neurite sprouts (NCS1, red; GFP, green; DAPI, blue; neurites from cell body, arrowhead; sprouts from longest neurite, arrows). (M–N) Quantification data show NCS1-transduced neurons (red bar) have a larger number of neurites from cell body and sprouts from longest neurites compared to control HIV-GFP-transduced neurons (black bar). Data are expressed as mean ± SEM from 3 independent experiments. Scale bars: (A,B,D,E,G,H,J,K) 100 µm; (C,F,I,L) 20 µm.
(3.10 MB TIF)

Figure S2 **Further examples of NCS1 overexpression promoting neurite sprouting via Akt activation in vitro.** (A–D) Primary cultured adult cortical neurons transduced with HIV-GFP-NCS1 have high phospho-Akt levels and more extensive neurite sprouting when treated with vehicle (0.01% DMSO). (E–H) Control GFP-transduced neurons have low phospho-Akt levels and few neurite sprouts when treated with vehicle. Phospho-Akt, red; GFP, green; DAPI, blue. Inserts are higher magnification of corresponding panels. Scale bars: 50 µm.
(1.90 MB TIF)

Figure S3 **Further examples of NCS1-induced neurite sprouting reduced by inhibiting Akt activation in vitro.** (A–D) Primary cultured adult cortical neurons transduced with HIV-GFP-NCS1 have reduced phospho-Akt levels and fewer neurite sprouts in the presence of LY249002. (E–H) Control HIV-GFP-transduced neurons continue to express low phospho-Akt levels and fewer neurite sprouts in the presence of LY249002. Phospho-Akt, red; GFP, green; DAPI, blue. Inserts are higher magnification of corresponding panels. Scale bars: 50 µm.
(1.63 MB TIF)

Figure S4 **Schematic diagrams illustrating the design and sequences of the in vivo experimental studies.** The green circle and line indicate the lentiviral vector transduced neurons and GFP positive labelled axons and collaterals, respectively. The white circle and line indicate untransduced neurons and axons. The black circle and line indicate the CST, which when transected rostral to the pyramidal decussation leads to degeneration of the main dorsal CST on the opposite side and a corresponding CST-denervation of the spinal cord (black dashed line). (A) A 3 wk primed NCS1 overexpression in the transduced non-axotomized corticospinal neurons (CSN) is predicted to promote axon collateral sprouting on the same side of the intact CST and into the CST-denervated side of the spinal cord, but not in the control group (thin green lines). To establish if the functional recovery is dependent on collateral sprouting from the intact CST into the CST-denervated side, a second lesion of the intact pyramidal tract then causes CST-denervation of the postulated source of sprouting resulting in loss of the previously achieved recovery in behaviour and EMG activity (dashed green lines). (B) A 2 d delayed NCS1 overexpression in the transduced non-axotomized CSN after a pyramidotomy is predicted to promote axon collateral sprouting on the same side of the intact CST and into the CST-denervated side of the spinal cord, but not in the control group (thin green lines). (C) A 2 d delayed NCS1 overexpression in the transduced axotomized CSN after a pyramidotomy is predicted to promote axonal sprouting and regeneration into and around the lesion site at the medullary level, but not in the control group (thin green lines). (D) A 1 wk primed NCS1 overexpression in the transduced axotomized CSN after a pyramidotomy is predicted to prevent cell atrophy, but not in the control group.
(2.00 MB TIF)

Figure S5 **Assessment of the pyramidotomy lesion.** (A–B) Macroscopic images of a unilateral left pyramidal tract lesion rostral to the decussation in the lower medulla. The lesion (arrow) generates contralaterally a CST-denervated side of the spinal cord. (C–D) Photomicrographs of reactive astrocytes (GFAP immunopositive) demarcating the lesion site and the absence of PKCγ immunostaining caudal to the lesion (arrow). Arrowhead and arrow indicate the intact and lesioned pyramidal tract, respectively. (E–F) Macroscopic images from a rat containing a previous unilateral pyramidotomy (arrow) with a subsequent lesion of the intact pyramid (arrowhead) resulting in complete CST-denervation of both sides of the spinal cord. (G–H) Macroscopic images of a sham operated rat that received a unilateral pyramidal tract lesion in a subsequent operation (arrowhead). Higher magnification in panels B, F, and H are indicated by dashed boxes in panels A, E, and G, respectively. Scale bars: (A, E, G) 5 mm, (B–D, F, H) 1 mm.
(8.28 MB TIF)

Figure S6 **Complete transverse section of cervical spinal cord from a pyramidotomized control GFP-transduced rat.** (A–C) Rat transduced with the control HIV-GFP lentivector has GFP positive fibers present in the CST-innervated side of the spinal cord (CST (A), dorsal horn (B), ventral horn (C)). (D–F) In contrast, few GFP positive fibers were detected in the CST-denervated side of the spinal cord (Dorsal horn (D), intermediate laminae (E), ventral horn (F)). Scale bars: (A–F) 200 µm.
(5.70 MB TIF)

Figure S7 **Complete transverse section of cervical spinal cord from a pyramidotomized NCS1-transduced rat**. (A–C) Animal transduced with the HIV-GFP-NCS1 lentivector has GFP positive fibers present in the CST-innervated side of the spinal cord (CST (A), dorsal horn (B), and ventral horn (C)). (D–F) On the CST-denervated side of the spinal cord, extensive amounts of GFP positive fibers have crossed the midline with axon collaterals detected in the dorsal horn (D), intermediate laminae (E), and ventral horn (F). Scale bars: (A–F) 200 m.
(5.13 MB TIF)

Figure S8 **Confirmation of pyramidotomy lesion using PKCγ immunostaining.** (A–D) Photomicrographs show the presence of bilateral PKCγ immunostaining in the intact dorsal CST in sham rats (arrows). (E–L) Following a unilateral pyramidotomy, PKCγ immunostaining is only present in the intact dorsal CST (arrow) and absent in the lesioned dorsal CST (arrowhead) in rats injected with either the control (GFP) or NCS1 overexpressing lentivector. The numbers at bottom left indicate the rat identity from which the tissues were taken. Scale bars: 200 µm.
(3.32 MB TIF)

Figure S9 **Unilateral pyramidotomy does not affect the CST-innervated limbs in behavioural tasks.** (A–B) In the staircase reaching test, the CST-innervated forelimb for both transduced groups showed no significant reduction in numbers of food pellets displaced or eaten compared to sham group following unilateral pyramidotomy. (C–D) In the grid exploration test, the CST-innervated fore- and hindlimbs showed no significant difference in the number of misplacements compared to the sham operated group following unilateral pyramidotomy. (A–D) One week after a subsequent operation to lesion the intact pyramidal tract, the previously CST-innervated forelimb showed a reduced capability to displace or to eat food pellets in the staircase reaching test, and the number of misplacements in the grid exploration test was increased. Black line, CST-innervated limb of GFP rats; red line, CST-innervated limb of NCS1 rats; blue line, limb of sham rats.
(0.37 MB TIF)

Figure S10 **The effects of increasing the number of stimulating pulses to the NCS1-transduced sensorimotor cortex on the EMG activities of the forelimbs.** (A–B) With 1 pulse, neither movement nor little or no EMG activity was observed in either forelimbs (ripple is attributed to low threshold neck muscle activity). (C–D) With 2 pulses, forelimb movements occurred and EMG responses were observed in both forelimbs, with delayed latency on the CST-denervated side. (E–H) With 3 or 4 pulses, the EMG responses further increased in size in both forelimbs. (I–J) After transection of the intact dorsal CST at C4 spinal level, the EMG response from the forelimb on the now acutely CST-denervated side was all but abolished and it was lost altogether from the chronically CST-denervated forelimb.
(0.59 MB TIF)

## Abbreviations

CMV: cytomegalovirus
CSN: corticospinal neurons
CST: corticospinal tract
DRG: dorsal root ganglion
GFP: green fluorescent protein
MAP2: microtubule-associated protein 2
NCS1: neuronal calcium sensor-1
PBS: phosphate-buffered saline
SCI: spinal cord injury
SFFV: spleen focus-forming virus
