# Supplementary figures and images for: Cortical Overexpression of Neuronal Calcium Sensor-1 Induces Functional Plasticity in Spinal Cord Following Unilateral Pyramidal Tract Injury in Rat
Source: PLoS Biol. 2010 Jun 22;8(6):e1000399. doi: 10.1371/journal.pbio.1000399 (PMC2889931; doi:10.1371/journal.pbio.1000399)

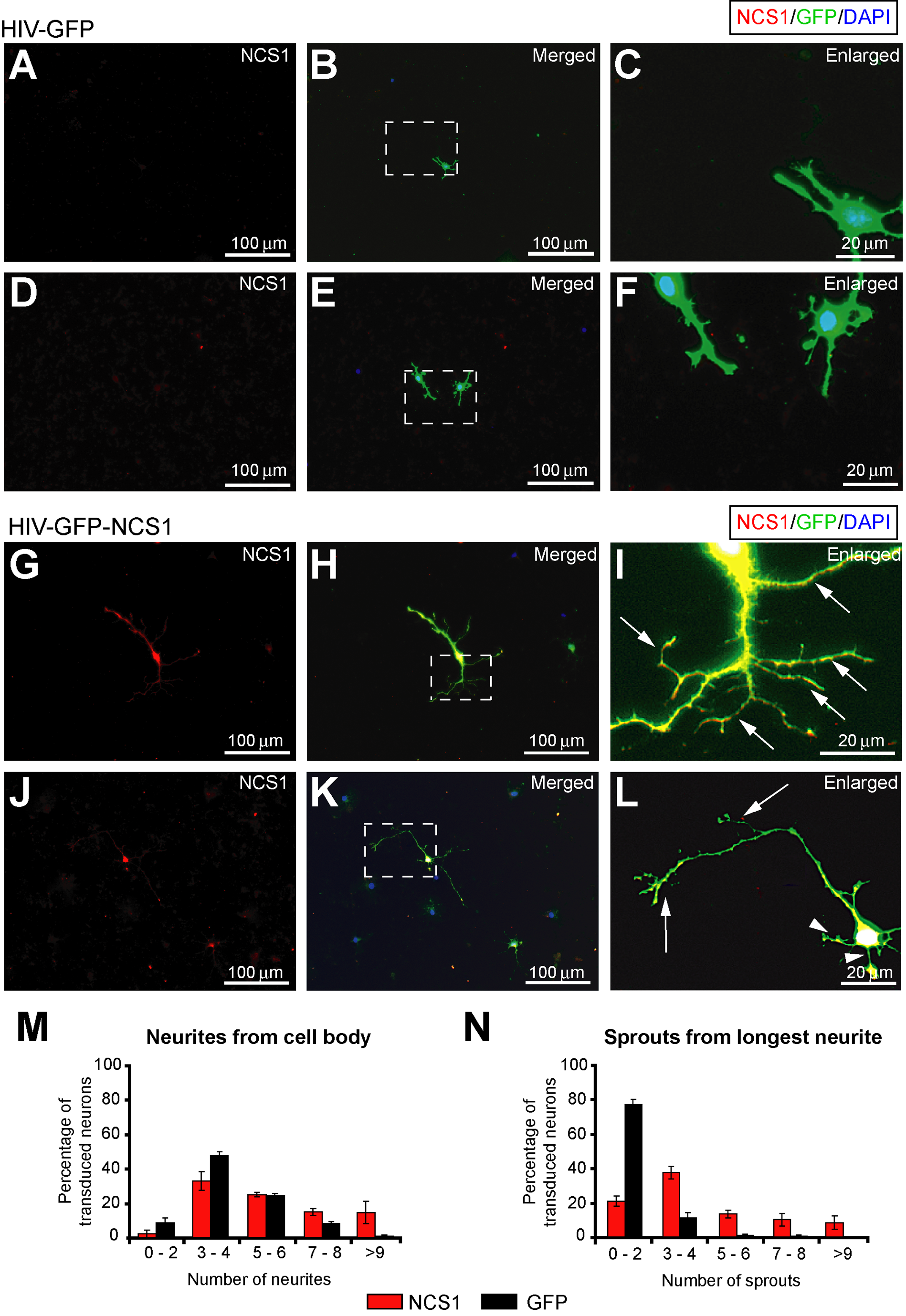

Supplement: Figure S1 — Further examples of NCS1 overexpression promoting neurite sprouting in vitro. (A–F) Primary cultured adult cortical neurons transduced with control HIV-GFP lentivector showing low levels of NCS1 immunostaining and limited neurite sprouts after 3 d in vitro. (G–L) Neurons transduced with HIV-GFP-NCS1 have higher levels of NCS1 immunostaining and a greater number of neurite sprouts (NCS1, red; GFP, green; DAPI, blue; neurites from cell body, arrowhead; sprouts from longest neurite, arrows). (M–N) Quantification data show NCS1-transduced neurons (red bar) have a larger number of neurites from cell body and sprouts from longest neurites compared to control HIV-GFP-transduced neurons (black bar). Data are expressed as mean ± SEM from 3 independent experiments. Scale bars: (A,B,D,E,G,H,J,K) 100 µm; (C,F,I,L) 20 µm. (3.10 MB TIF) [file pbio.1000399.s001.tif]

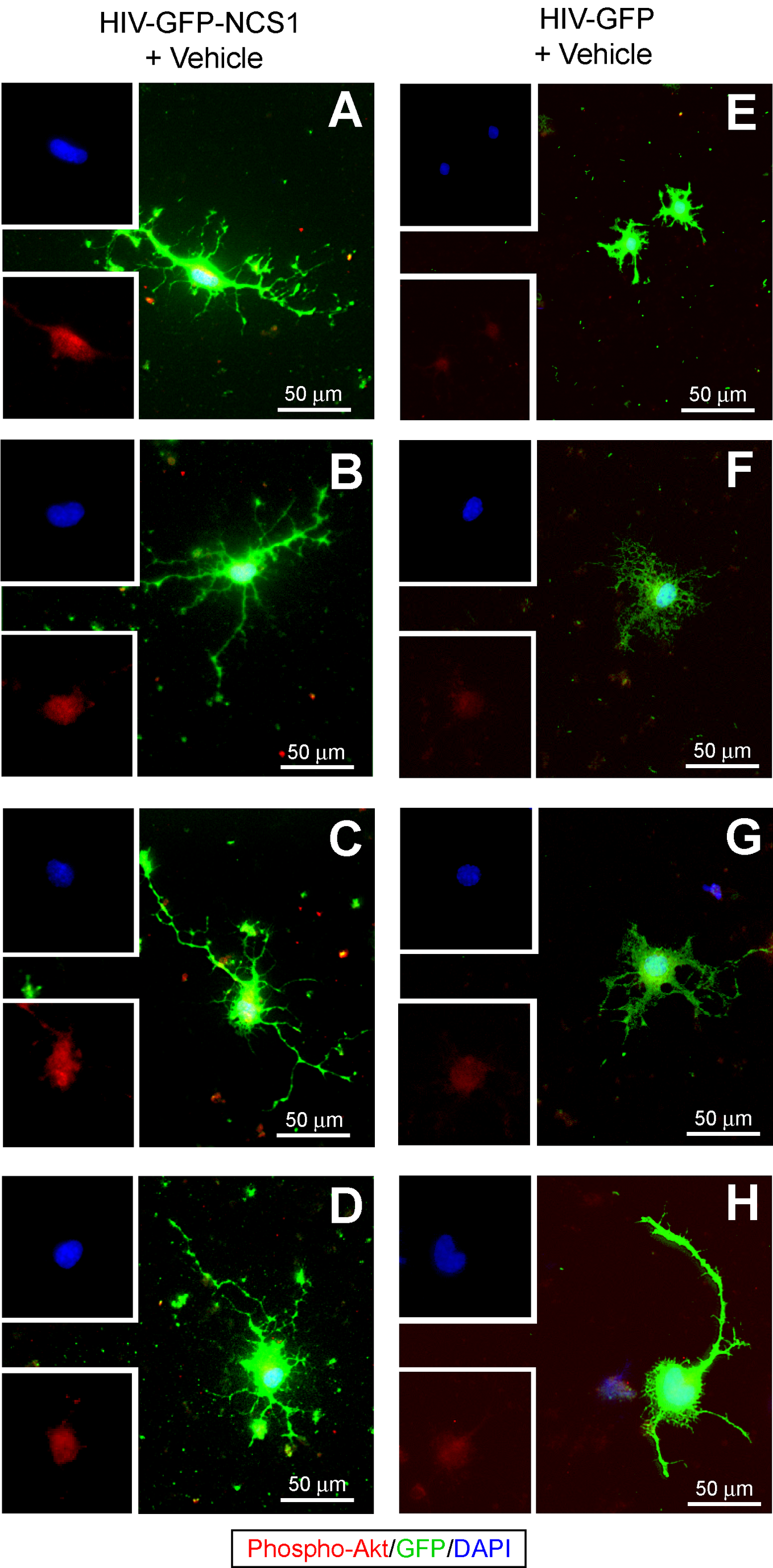

Supplement: Figure S2 — Further examples of NCS1 overexpression promoting neurite sprouting via Akt activation in vitro. (A–D) Primary cultured adult cortical neurons transduced with HIV-GFP-NCS1 have high phospho-Akt levels and more extensive neurite sprouting when treated with vehicle (0.01% DMSO). (E–H) Control GFP-transduced neurons have low phospho-Akt levels and few neurite sprouts when treated with vehicle. Phospho-Akt, red; GFP, green; DAPI, blue. Inserts are higher magnification of corresponding panels. Scale bars: 50 µm. (1.90 MB TIF) [file pbio.1000399.s002.tif]

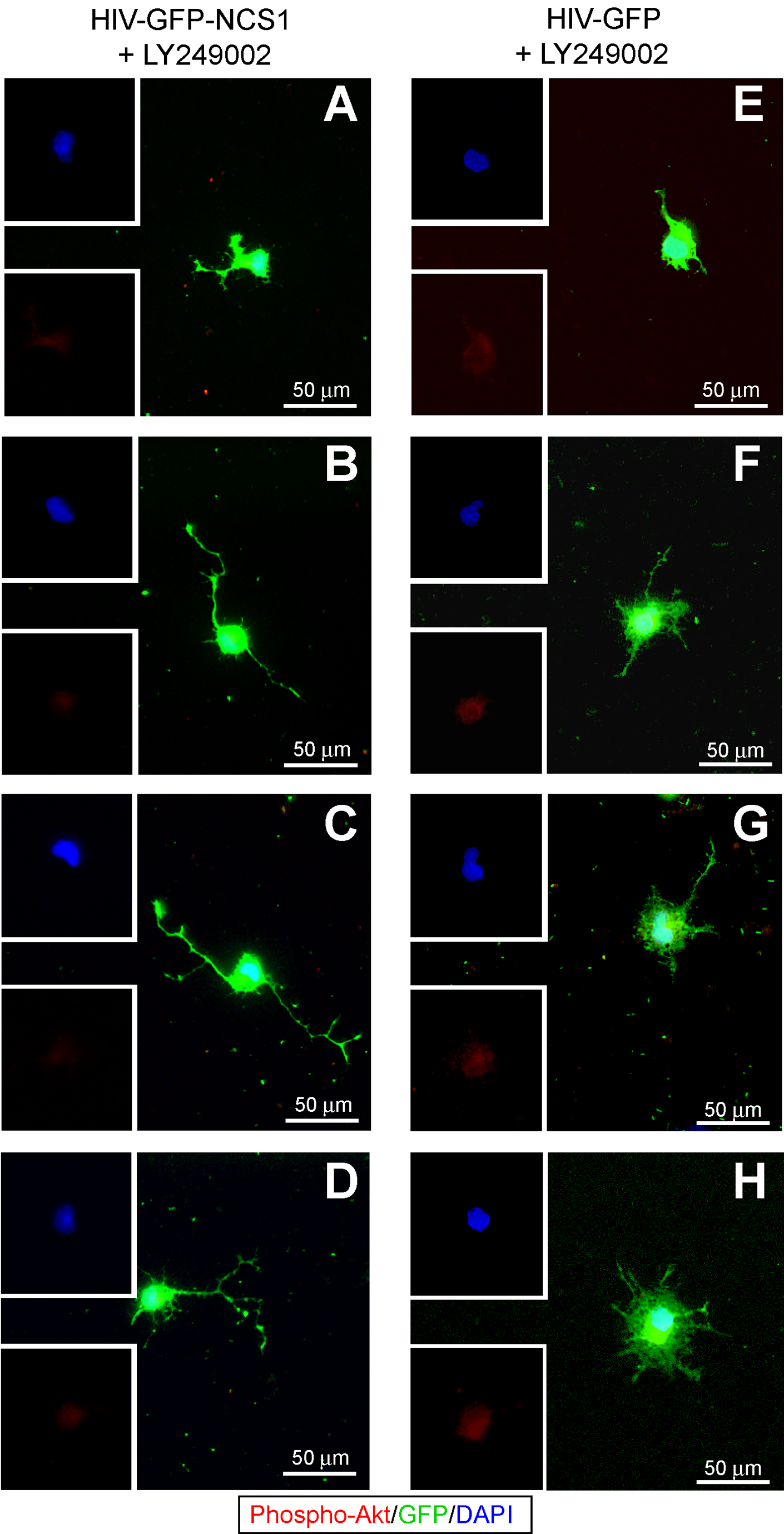

Supplement: Figure S3 — Further examples of NCS1-induced neurite sprouting reduced by inhibiting Akt activation in vitro. (A–D) Primary cultured adult cortical neurons transduced with HIV-GFP-NCS1 have reduced phospho-Akt levels and fewer neurite sprouts in the presence of LY249002. (E–H) Control HIV-GFP-transduced neurons continue to express low phospho-Akt levels and fewer neurite sprouts in the presence of LY249002. Phospho-Akt, red; GFP, green; DAPI, blue. Inserts are higher magnification of corresponding panels. Scale bars: 50 µm. (1.63 MB TIF) [file pbio.1000399.s003.tif]

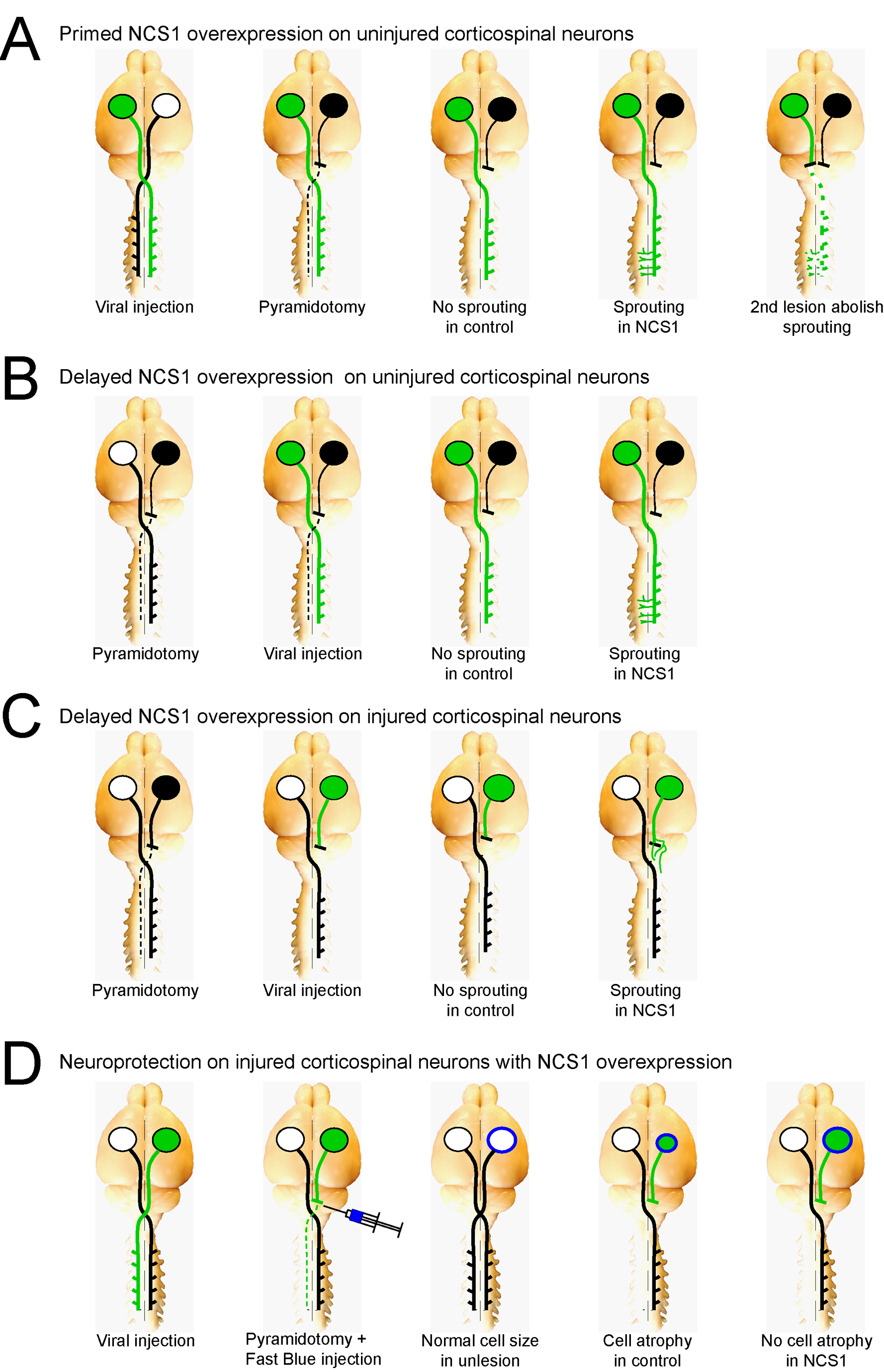

Supplement: Figure S4 — Schematic diagrams illustrating the design and sequences of the in vivo experimental studies. The green circle and line indicate the lentiviral vector transduced neurons and GFP positive labelled axons and collaterals, respectively. The white circle and line indicate untransduced neurons and axons. The black circle and line indicate the CST, which when transected rostral to the pyramidal decussation leads to degeneration of the main dorsal CST on the opposite side and a corresponding CST-denervation of the spinal cord (black dashed line). (A) A 3 wk primed NCS1 overexpression in the transduced non-axotomized corticospinal neurons (CSN) is predicted to promote axon collateral sprouting on the same side of the intact CST and into the CST-denervated side of the spinal cord, but not in the control group (thin green lines). To establish if the functional recovery is dependent on collateral sprouting from the intact CST into the CST-denervated side, a second lesion of the intact pyramidal tract then causes CST-denervation of the postulated source of sprouting resulting in loss of the previously achieved recovery in behaviour and EMG activity (dashed green lines). (B) A 2 d delayed NCS1 overexpression in the transduced non-axotomized CSN after a pyramidotomy is predicted to promote axon collateral sprouting on the same side of the intact CST and into the CST-denervated side of the spinal cord, but not in the control group (thin green lines). (C) A 2 d delayed NCS1 overexpression in the transduced axotomized CSN after a pyramidotomy is predicted to promote axonal sprouting and regeneration into and around the lesion site at the medullary level, but not in the control group (thin green lines). (D) A 1 wk primed NCS1 overexpression in the transduced axotomized CSN after a pyramidotomy is predicted to prevent cell atrophy, but not in the control group. (2.00 MB TIF) [file pbio.1000399.s004.tif]

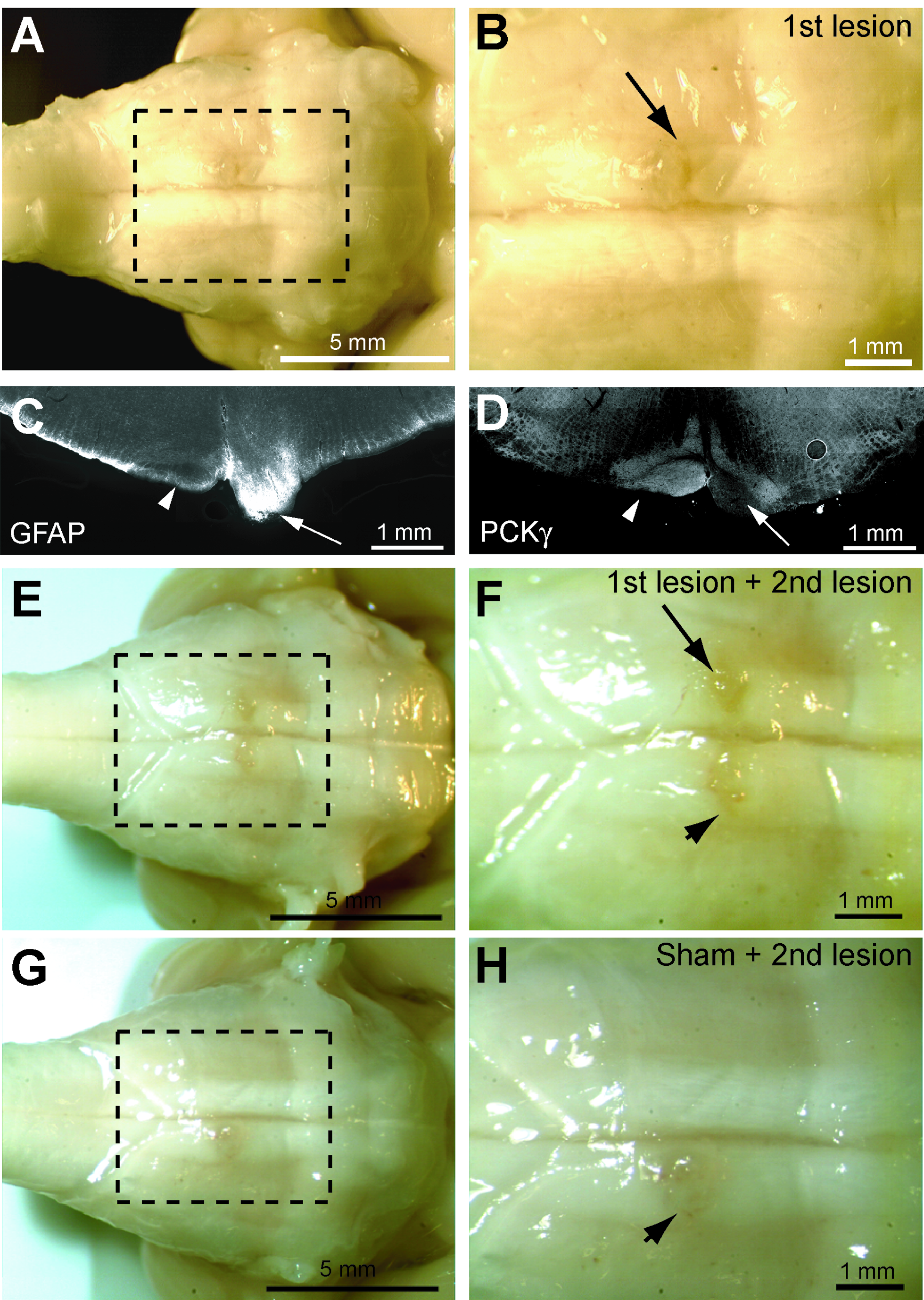

Supplement: Figure S5 — Assessment of the pyramidotomy lesion. (A–B) Macroscopic images of a unilateral left pyramidal tract lesion rostral to the decussation in the lower medulla. The lesion (arrow) generates contralaterally a CST-denervated side of the spinal cord. (C–D) Photomicrographs of reactive astrocytes (GFAP immunopositive) demarcating the lesion site and the absence of PKCγ immunostaining caudal to the lesion (arrow). Arrowhead and arrow indicate the intact and lesioned pyramidal tract, respectively. (E–F) Macroscopic images from a rat containing a previous unilateral pyramidotomy (arrow) with a subsequent lesion of the intact pyramid (arrowhead) resulting in complete CST-denervation of both sides of the spinal cord. (G–H) Macroscopic images of a sham operated rat that received a unilateral pyramidal tract lesion in a subsequent operation (arrowhead). Higher magnification in panels B, F, and H are indicated by dashed boxes in panels A, E, and G, respectively. Scale bars: (A, E, G) 5 mm, (B–D, F, H) 1 mm. (8.28 MB TIF) [file pbio.1000399.s005.tif]

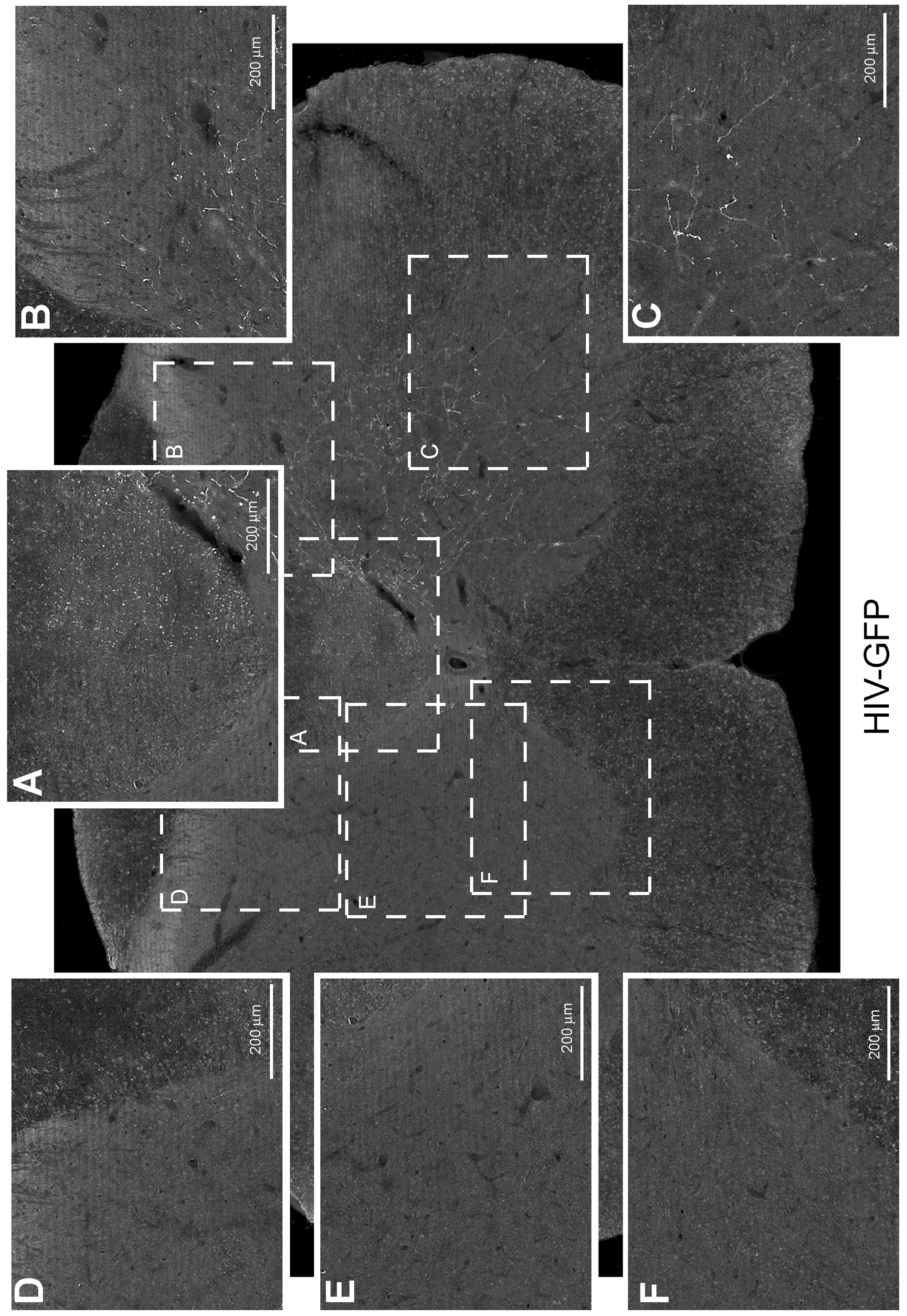

Supplement: Figure S6 — Complete transverse section of cervical spinal cord from a pyramidotomized control GFP-transduced rat. (A–C) Rat transduced with the control HIV-GFP lentivector has GFP positive fibers present in the CST-innervated side of the spinal cord (CST (A), dorsal horn (B), ventral horn (C)). (D–F) In contrast, few GFP positive fibers were detected in the CST-denervated side of the spinal cord (Dorsal horn (D), intermediate laminae (E), ventral horn (F)). Scale bars: (A–F) 200 µm. (5.70 MB TIF) [file pbio.1000399.s006.tif]

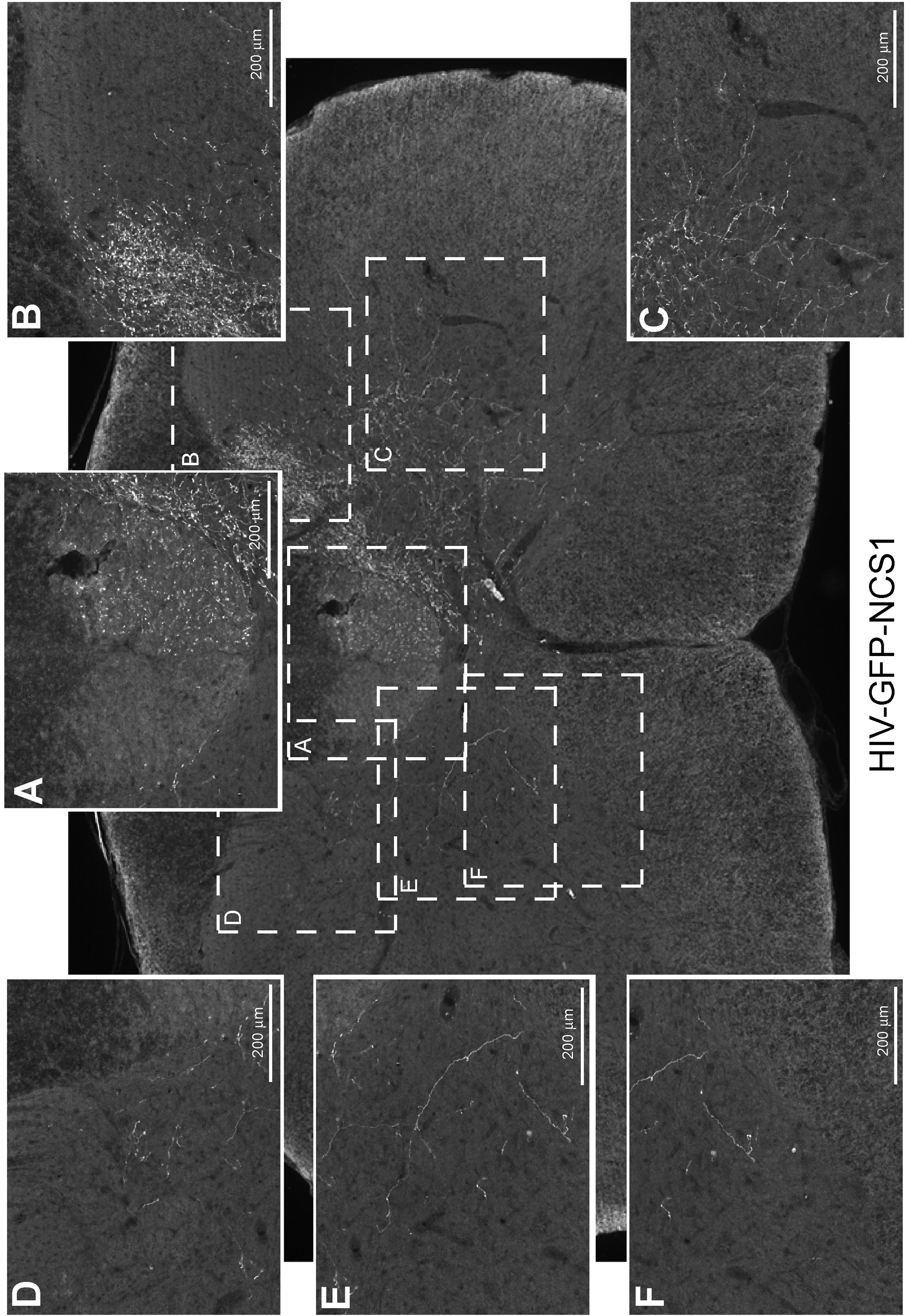

Supplement: Figure S7 — Complete transverse section of cervical spinal cord from a pyramidotomized NCS1-transduced rat. (A–C) Animal transduced with the HIV-GFP-NCS1 lentivector has GFP positive fibers present in the CST-innervated side of the spinal cord (CST (A), dorsal horn (B), and ventral horn (C)). (D–F) On the CST-denervated side of the spinal cord, extensive amounts of GFP positive fibers have crossed the midline with axon collaterals detected in the dorsal horn (D), intermediate laminae (E), and ventral horn (F). Scale bars: (A–F) 200 m. (5.13 MB TIF) [file pbio.1000399.s007.tif]

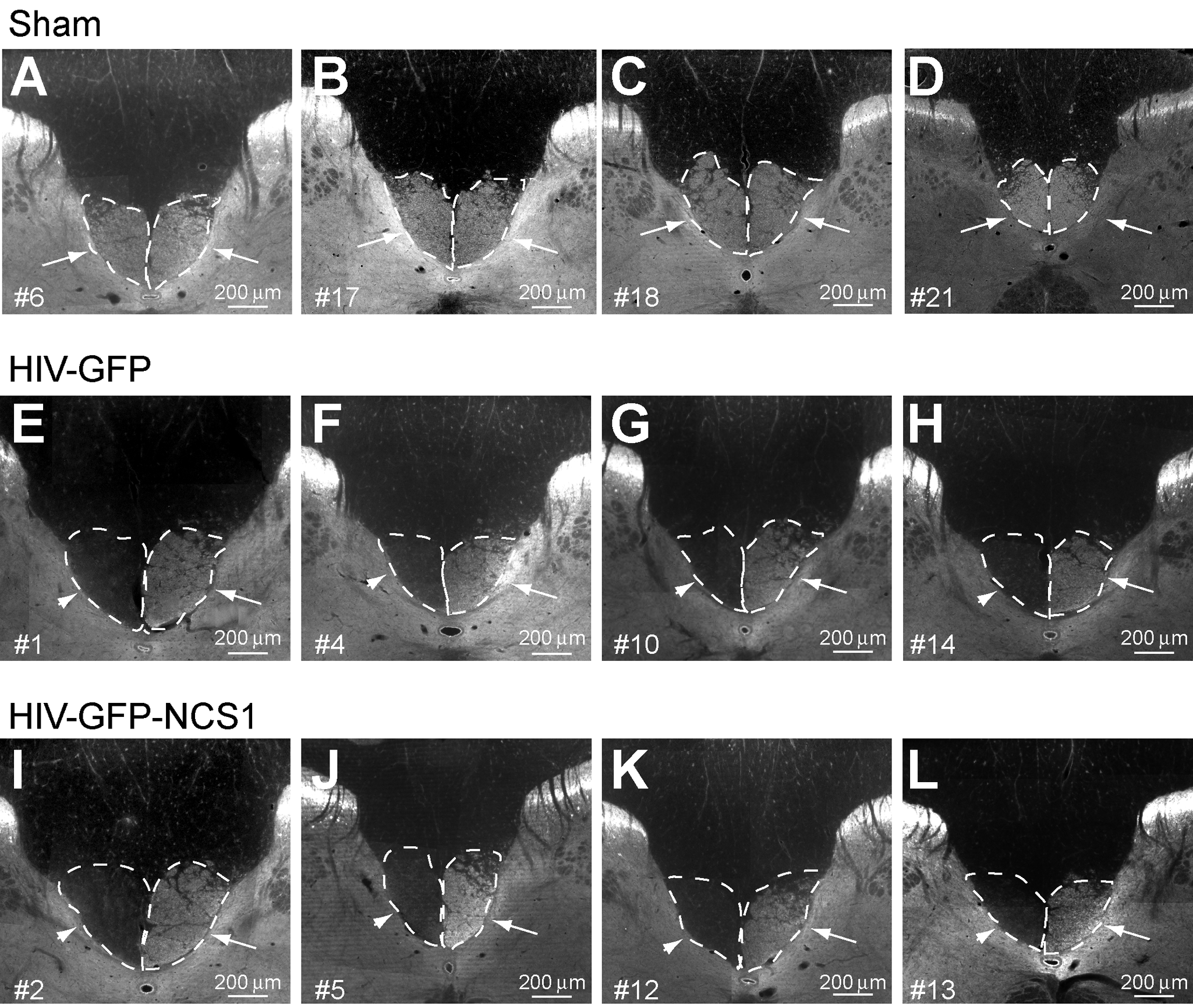

Supplement: Figure S8 — Confirmation of pyramidotomy lesion using PKCγ immunostaining. (A–D) Photomicrographs show the presence of bilateral PKCγ immunostaining in the intact dorsal CST in sham rats (arrows). (E–L) Following a unilateral pyramidotomy, PKCγ immunostaining is only present in the intact dorsal CST (arrow) and absent in the lesioned dorsal CST (arrowhead) in rats injected with either the control (GFP) or NCS1 overexpressing lentivector. The numbers at bottom left indicate the rat identity from which the tissues were taken. Scale bars: 200 µm. (3.32 MB TIF) [file pbio.1000399.s008.tif]

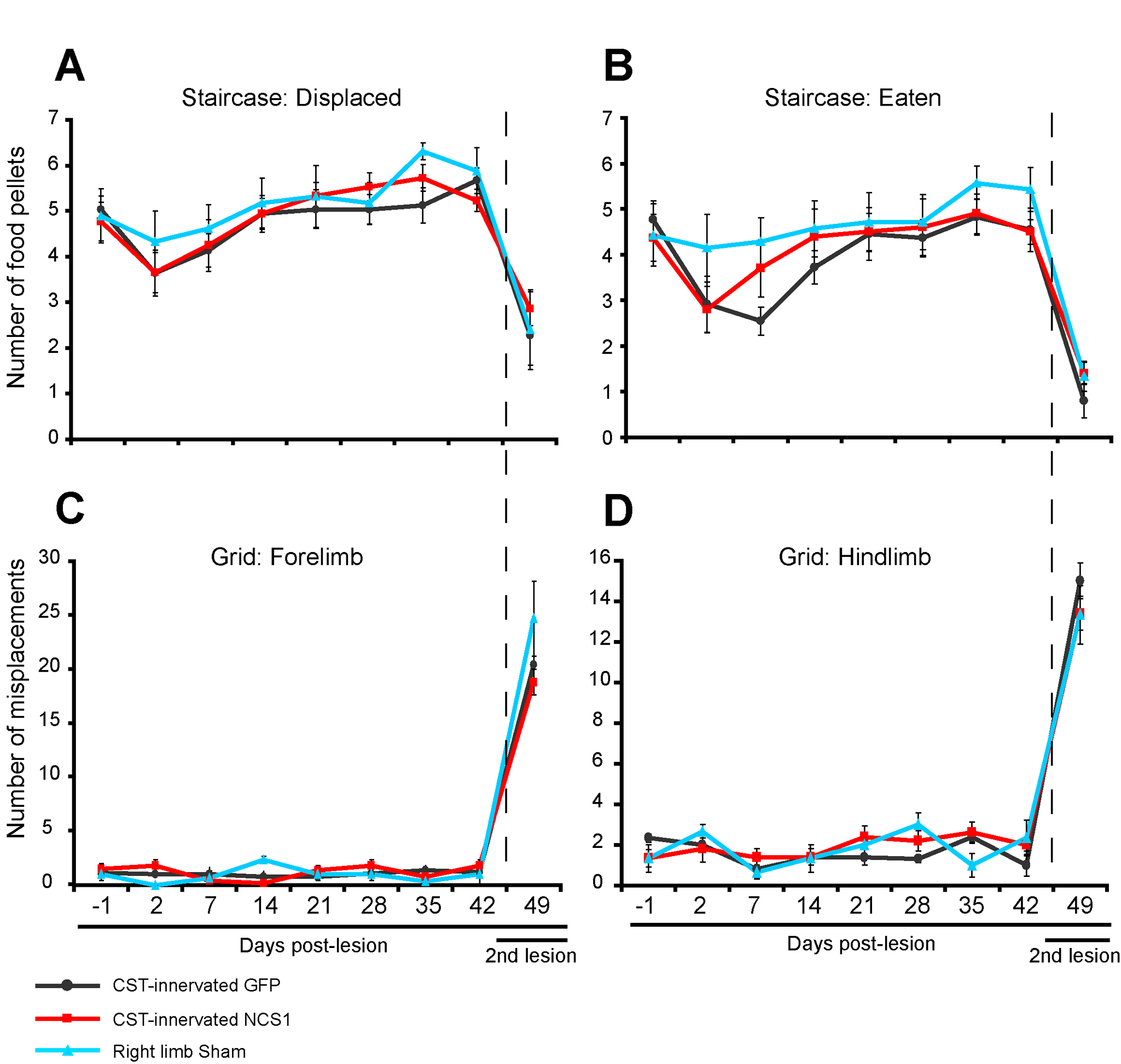

Supplement: Figure S9 — Unilateral pyramidotomy does not affect the CST-innervated limbs in behavioural tasks. (A–B) In the staircase reaching test, the CST-innervated forelimb for both transduced groups showed no significant reduction in numbers of food pellets displaced or eaten compared to sham group following unilateral pyramidotomy. (C–D) In the grid exploration test, the CST-innervated fore- and hindlimbs showed no significant difference in the number of misplacements compared to the sham operated group following unilateral pyramidotomy. (A–D) One week after a subsequent operation to lesion the intact pyramidal tract, the previously CST-innervated forelimb showed a reduced capability to displace or to eat food pellets in the staircase reaching test, and the number of misplacements in the grid exploration test was increased. Black line, CST-innervated limb of GFP rats; red line, CST-innervated limb of NCS1 rats; blue line, limb of sham rats. (0.37 MB TIF) [file pbio.1000399.s009.tif]

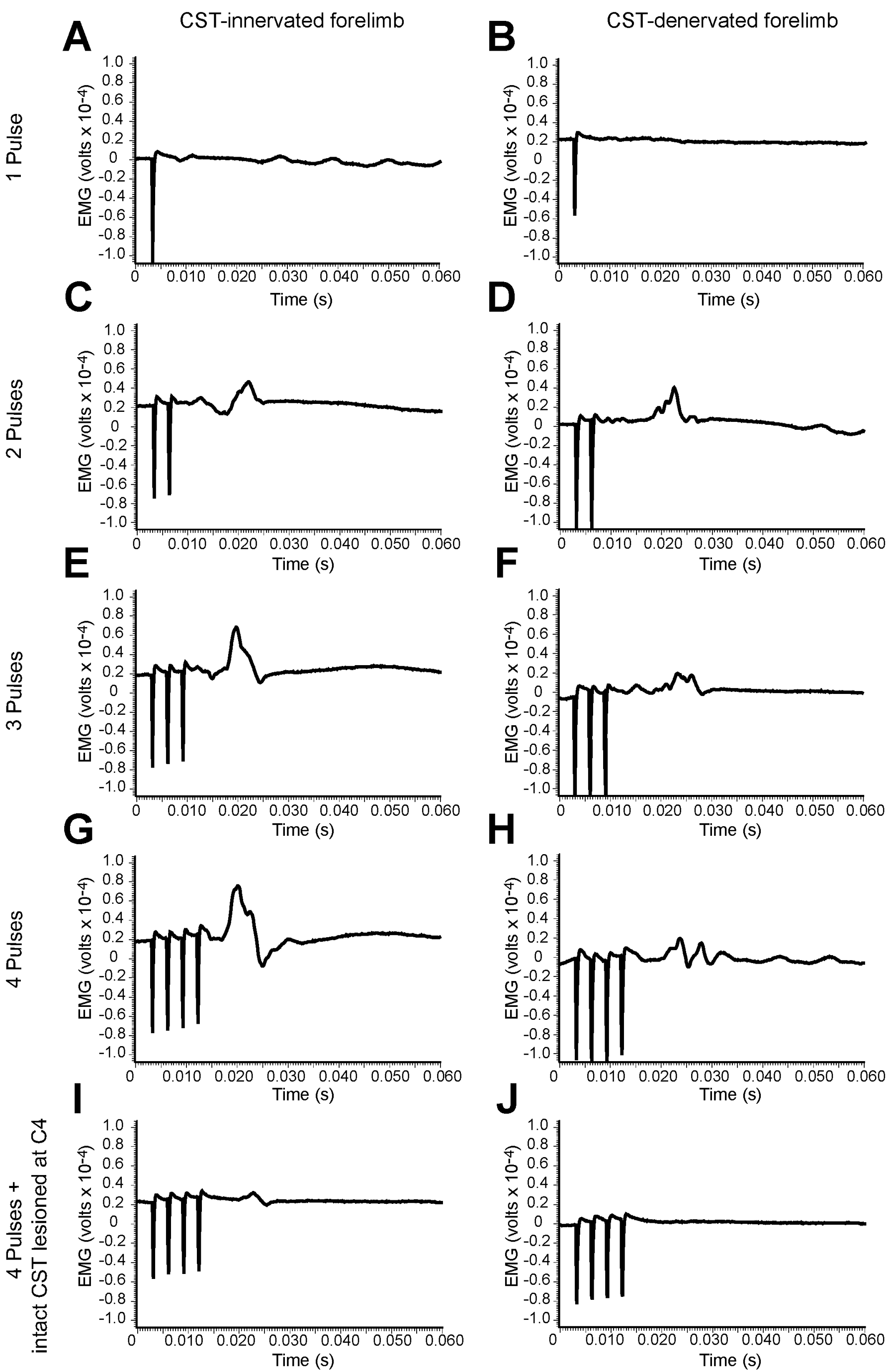

Supplement: Figure S10 — The effects of increasing the number of stimulating pulses to the NCS1-transduced sensorimotor cortex on the EMG activities of the forelimbs. (A–B) With 1 pulse, neither movement nor little or no EMG activity was observed in either forelimbs (ripple is attributed to low threshold neck muscle activity). (C–D) With 2 pulses, forelimb movements occurred and EMG responses were observed in both forelimbs, with delayed latency on the CST-denervated side. (E–H) With 3 or 4 pulses, the EMG responses further increased in size in both forelimbs. (I–J) After transection of the intact dorsal CST at C4 spinal level, the EMG response from the forelimb on the now acutely CST-denervated side was all but abolished and it was lost altogether from the chronically CST-denervated forelimb. (0.59 MB TIF) [file pbio.1000399.s010.tif]
